# Ecology of endolithic bryozoans: colony development, growth rates and interactions of species in the genus *Immergentia*

**DOI:** 10.1186/s40851-024-00246-9

**Published:** 2024-12-31

**Authors:** Mildred J. Johnson, Sarah Lemer, Masato Hirose, Sebastian H. Decker, Thomas Schwaha

**Affiliations:** 1 Dept. Evolutionary Biology, University of Vienna, Djerassiplatz 1, Vienna, 1030 Austria; 2 Marine Laboratory, UOG Station, Mangilao Guam, 96923 USA; 3 Leibniz Institute for the Analysis of Biodiversity Change, Museum of Nature, Hamburg, 20146, Germany; 4 School of Marine Biosciences, Kitasato University, Kitasato 1-15-1, Sagamihara-Minami, Kanagawa, 252-0373 Japan

**Keywords:** Ancestrula, Zooids, Colony density, Boring bryozoan, Astogeny, Distribution

## Abstract

Boring bryozoans dissolve calcium carbonate substrates, leaving unique borehole traces. Depending on the shell type, borehole apertures and colony morphology can be diagnostic for distinguishing taxa, but to discriminate among species their combination with zooidal morphology is essential. All boring (endolithic) bryozoans are ctenostomes that, along with other boring taxa, are common in benthic communities. The growth rates of such bryozoans, including Immergentiidae, are largely unknown. For the first time laboratory experiments were conducted to determine growth rates and early colony development of the intertidal species *Immergentia stephanieae* and the subtidal species *I.* cf. *suecica* from Roscoff, France. In growth experiment 1, ancestrular growth rates varied, with the highest rates in *I. stephanieae* at 96.5 µm day^−1^ and the lowest at 1.1 µm day^−1^, during the period of August to October, in which the number of reproductive zooids was comparably higher than in other months of the year. *Immergentia* cf. *suecica* had a higher proportion of reproductive zooids from December to March compared to other months. In growth experiment 2, the bryozoans were fed a culture mixture of *Chaetoceros calcitrans* and *Tisochrysis lutea* which was compared with a control. The growth rate of small colonies of comparable size was greater in the food-enriched samples compared to the control (non-enriched). In larger colonies, the trend differed with greater growth (cystid appendage expansion) rate reported for some samples in the control. In food-enriched samples ancestrulae of *I. stephanieae* grew at 23 µm day^−1^ and *I.* cf. *suecica* 9.3 µm day^−1^ while no growth was observed in the control of *I.* cf. *suecica*, but 0.4 µm day^−1^ was reported for *I. stephanieae*. Growth patterns in the early developmental stages showed that the budding patterns from the ancestrulae were the same for both species, with different enantiomorphic tendencies. Inter- and intraspecific interactions are also discussed. The distribution of immergentiids is presented, as are records from new locations and the greatest subtidal depth of collection reported to date.

**Supplementary Information:**

The online version contains supplementary material available at 10.1186/s40851-024-00246-9.

## Background

Bryozoans are a phylum of sessile, colonial suspension feeders [1], with the exception of a few solitary forms [2–4]. They are classified into two clades, Phylactolaemata and Myolaemata, with the latter comprising Stenolaemata and Gymnolaemata [5]. Within Gymnolaemata, Ctenostomata is a paraphyletic group of gymnolaemates [6] characterized by an uncalcified cuticle. The Cheilostomata, in contrast, are characterized by a calcified body wall and originated from ctenostome-like ancestors. To date, close to 400 species of ctenostomes have been described [7] compared to cheilostomes with over 6000 extant species [8]. The Immergentiidae constitute one of the four families of extant endolithic ctenostomes, the so-called boring bryozoans, owing to their ability to dissolve calcium carbonate by chemical means [9, 10]. The other endolithic ctenostome families are Penetrantiidae, Spathiporidae and Terebriporidae. Altogether, fewer than 50 extant boring bryozoan species have been described.

Generally, new bryozoan colonies are formed by free-swimming sexually produced larvae that undergo metamorphosis after settlement on a substrate. The ancestrula, the founding zooid, develops during metamorphosis [11, 12] and propagates by asexual budding [13, 14]. This results in genetically identical modules called zooids that form a colony [1]. Each zooid consists of a cystid (the protective body wall) and a retractable polypide consisting of a lophophore, a U-shaped digestive tract, and associated neural and muscular systems. Zooids can be polymorphic and differ in appearance and function (heterozooids) compared to regular feeding autozooids of similar shape and size. Marine bryozoan colonies can consist of sterile and sexual zooids (gonochoric: male and/or female) or hermaphrodites [11, 15]. In gymnolaemates, two categories of reproductive strategies are recognized; namely, zygote spawning (no incubation) and brooding (see [12, 16–18]). In both strategies, sperm is released into the water column, and internal fertilization occurs [15], however, in spawning species, zygotes are released into the water column after fertilization, and the embryo develops into a long-lived planktotrophic cyphonautes larva (see [18, 19]). In brooding species, zygotes are retained and the embryo develops into a short-lived lecithotrophic coronate larva (see [15, 20, 21]). In immergentiids, spathiporids, and penetrantiids, a single embryo is brooded. In immergentiids and spathiporids the embryo develops in the tentacle sheath of an autozooid with a degenerated polypide [9, 22, 23] and in a gonozooid in penetrantiids [9, 24].

The number and orientation of cystid appendages and stolons emerging from ancestrulae has been analysed in the early development of boring ctenostomes, and whether this aspect can be diagnostic for species identification has been discussed [10]. Early bud formation and development has been studied in immergentiids and penetrantiids, especially *Penetrantia densa* [9]. Subsequent descriptions of colony development are primarily based on the external colony morphology of established colonies (see [10, 25, 26]). Published data on growth experiments and development of boring bryozoans are lacking, while more findings have been reported on other bryozoans, predominantly cheilostomes. Growth experiments have been conducted with a variety of treatments and variables, such as feeding cultures and preferences [27–30], flow and feeding rates [31–35], temperature [13, 36], interactions with other bryozoans and colony densities [37] or ocean acidification scenarios [38–40]. Several reviews on various feeding regimes and growth forms have been published recently (see [41–44]). Marine bryozoans mostly feed on phytoplankton [1, 41, 45] and their feeding mechanisms are well established. Bryozoans themselves are a food source for other marine organisms (see [46, 47]) but information on inter- and intraspecies interactions of boring bryozoans is rare.

Although little is known about the distribution of boring bryozoans, data on the locations of representative species indicate that these organisms occur in temperate and tropical marine regions at various depths, but not in polar regions. For example, extant species have been reported in the North Atlantic [9, 23, 48, 49], South Atlantic [10, 50], the Mediterranean Sea [51, 52], North Pacific [26, 50, 53] and South Pacific [23, 48, 50]. The distribution of penetrantiids was recently summarized by Decker et al. [24].

To enhance our understanding of the life histories of boring bryozoans, we examined colony development by (1) conducting settlement experiments and observing early growth patterns, (2) determining and comparing growth rates in *Immergentia stephanieae* Johnson & Schwaha 2024 (intertidal species) and *Immergentia* cf. *suecica* Silén 1947 (subtidal species) with or without supplemented food culture, (3) observing feeding behaviors, (4) evaluating the implications of species interactions on the substrate, and (5) updating the geographic distribution of immergentiids.

## Methodology

### Sampling sites and distribution

**Fig. 1:**
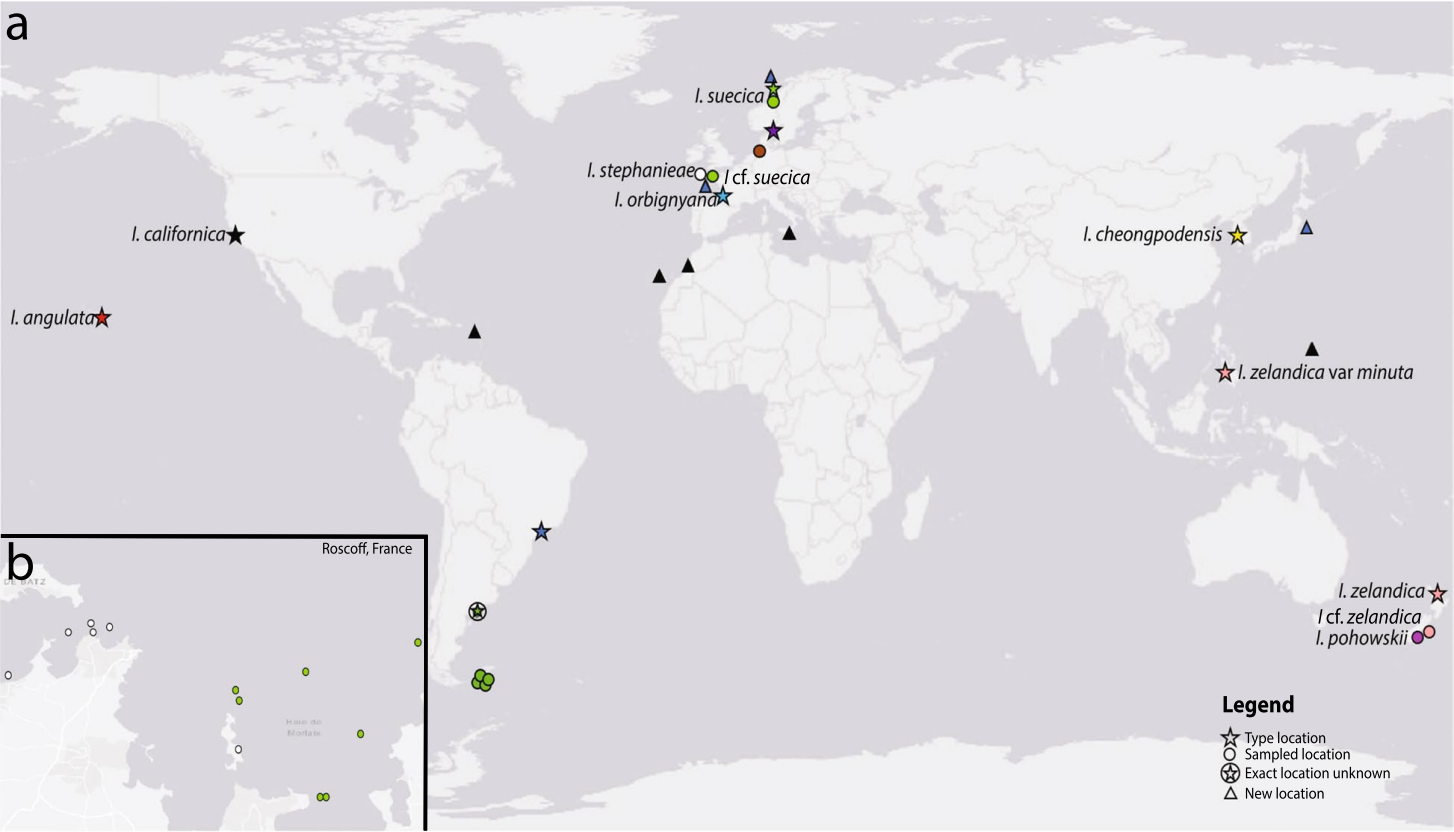
Distribution of immergentiids. **a** Type species of *Immergentia* indicated by star and species collected from different locations indicated by a circle. *Immergentia patagoniana* (dark green) in the Southwest Atlantic Ocean, *Immergentia stephanieae* (white) and *Immergentia* cf. *suecica* (light green) from the Northeast Atlantic, *Immergentia pohowskii* (magenta) and *Immergentia* cf. *zelandica* (pink) from the South Pacific. Immergentiid traces from new locations with known coordinates of sample sites along the west coast of Japan, Family Beach and Pago Bay (Guam), Bay of Biscay (France) and Ramfjorden (Norway) in dark blue triangles, new locations without known coordinates in black triangles from the Caribbean Sea (Guadeloupe), Agadir (Morocco), Tenerife (Spain), Syracuse (Italy). *Immergentia* spp. from Helgoland in brown. **b** Intertidal and subtidal locations of *Immergentia stephanieae* (white) and *Immergentia* cf. *suecica* (light green) in and off the coast of Roscoff France

Shells containing immergentiids were either collected by hand from intertidal zones or dredged (subtidal). Sample collection data was obtained from Burdwood Bank in the southwest Atlantic Ocean, from southern New Zealand and Trondheim Fjord, Norway (see [23]), as well as Pago Bay and Family Beach in Guam, USA; Helgoland, Germany and Sagami Bay and Ise Bay area (Enshu Sea) Japan (Fig. 1a). Additionally, molluscan shell and cold-water coral collections were examined for immergentiid borehole apertures at the Senckenberg am Meer Institute in Wilhelmshaven, Germany. Molluscan substrates not previously reported are also included here, along with the state of substrate (Table S1).

For laboratory experiments, the main study site was Roscoff in France where *Immergentia stephanieae* (intertidal) and *Immergentia* cf. *suecica* (subtidal) were sampled from several locations (Fig. 1b).

Location data from this study was combined with that of type specimens from literature and a distribution map was created with QGIS 3.32.2 Lima and edited with Adobe Photoshop (Adobe Inc.).

### Decalcification and whole mounts

Shells with immergentiid borings were decalcified in 20% ethylenediaminetetraacetic acid (EDTA) (pH 8.3). Samples were then washed three times in a 0.1 M phosphate buffer (PB) (pH 7.3) with 15-min intervals between washes. Zooids extracted from the extracellular matrix of the shell were stored in PB for immediate analysis or in 0.1 M PB with 0.1% sodium azide (NaN_3_) at 4 °C for extended periods. Images of immergentiids in substrate were produced with a Nikon SMZ800 (Nikon, Tokyo, Japan) stereomicroscope equipped with a Nikon Z6 camera and/or a Nikon SMZ25 stereomicroscope equipped with a DsRi2 camera. Images of mounted zooids were captured with a Nikon NiU compound microscope fitted with a DsRi2. Histology was based on established methods (see [23]).

### Immunocytochemical staining and confocal laser scanning microscopy

Specimens for immunochemical staining were fixed in 4% paraformaldehyde in phosphate buffer (PB) for two hours at room temperature, followed by three rinses of 20 min each in PB. Following decalcification and washing as described above, samples were treated with a solution of 2% Triton X-100 and 2% DMSO in phosphate buffer (PBT) overnight to increase tissue permeability. The samples were incubated overnight at room temperature in primary antibodies against acetylated alpha-tubulin raised in mouse (Sigma Aldrich, St. Louis, MO, USA) diluted in PB (concentration of 1:800). After washing three times with PB, samples were placed in a mixture consisting of the secondary antibody AlexaFluor 568 (Invitrogen, Carlsbad, CA, USA; concentration of 1:300, raised in goat against mouse), AlexaFluor 488 phalloidin (Invitrogen, Carlsbad, CA, USA; concentration of 1:100) for f-actin staining, and DAPI at a concentration of 1:100 in PB, for cell nuclei staining. The next day samples were washed and mounted on microscope slides with Flouromount G (Southern Biotec, Birmingham, LA, USA) and refrigerated at 4 °C. Mounted samples were scanned with a Leica SP5II confocal laser scanning microscope (Leica Microsystems, Wetzlar, Germany) and afterwards visualized with Amira version 2020.2 (Thermo Scientific™).

### Scanning electron microscopy

A dried gastropod shell with *I. stephanieae* borings was coated with gold in a JEOL JFC 2300 HR sputter coater (JEOL, Akishima, Tokyo, Japan) for 100 s. Images were acquired with the JEOL IT 300 scanning electron microscope (JEOL, Akishima, Tokyo, Japan), using secondary electron (LVSED) and backscatter electron detectors (BED-C) at 25 kV.

#### Sampling and feeding

Sampling and laboratory experiments were performed from 16 August to 16 October 2021 (growth and settlement experiment 1), and from 27 February to 22 March 2023 (growth and settlement experiment 2) at the Station Biologique De Roscoff (Roscoff Marine Station), France. Additional material collected in 2019, 2020, 2022 and 2023 was examined for indications of seasonality of species.

Cultures of the diatom *Chaetoceros calcitrans* and the haptophyte *Tisochrysis lutea* both approximately 5 µm in size were readily available at the research station. A mixture of both microalgae was used as a food supplement based on results of preliminary experiments.

### Experimental design: Growth and settlement experiments

#### Growth experiment 1

Small colonies (four-zooid-stage or less) and colonies with observable growing edges were photographed daily. Both categories of immergentiids were placed in separate 25 L aquaria supplied with unfiltered seawater and provided daily with 100 ml of *C. calcitrans* and *T. lutea,* under natural light conditions. The average water temperature in the aquaria was 16 °C during the course of the experiment (Fig. S1, S2a) ranging between 16 °C and 18 °C.

#### Growth experiment 2

Colonies of *I. stephanieae* were collected from the type locality (labelled T or TL), east of the research station (labelled RS) and colonies of *I.* cf. *suecica* from Stolvezen (labelled S). Colonies were grouped into two sizes; small colonies (four-zooid-stage or less, including ancestrulae) and large colonies (> 30 zooids) with observable growing edges. The substrates bearing these colonies were glued onto a 30 mm Petri dish with the quick drying MICROBE-LIFT® Coralscaper gel glue (Ecological Laboratories, USA; ARKA Biotechnologie GmbH, Germany) and the budding zones were photographed daily.

Generally, 25 L aquaria were set up so that colonies of both species received only unfiltered seawater (the control) or seawater with additional food in volumes of 50 ml of *C. calcitrans* and 50 ml of *T. lutea* (Fig. S1). Additional colonies were also kept under the same treatments as above and observed on a weekly basis. The average water temperature in the aquaria was 11 °C during the course of the experiment rarely ranging between 9 °C and 13 °C during the course of the day.

### Determination of growth

Measurements were based only on features visible on the substrate surface. Thus, the exact length of upward bending of secondary cystid appendages and exact stage of a developing zooid were difficult to ascertain. Completion of zooid formation was evident only when the borehole aperture broke through and probing or feeding commenced. Similarly, a bud was recognizable as a lateral swelling on the primary cystid appendage.

For small colonies the extension of cystid appendages (cya) was determined by subtracting the final length measurements (at the end of the experiment) from the initial length. For large colonies an additive length increase was used, that is the sum of up to four new branch lengths of the primary cystid appendages or lateral cystid appendages along the growing edge. In addition, the number of zooids or buds that developed were recorded. The growth rate was determined with the same equation.

Growth rate day^−1^ = final cya length – initial cya length / number of days observed.

Growth rate per day instead of raw values were used to compare colony growth because the total number of days of observation varied.

### Settlement experiment 1

Experiment 1A: A variety of molluscan shells were screened to detect any boring or epibenthic communities. Uncolonized shells of *Lutraria lutraria* and *Anomia ephippium* were selected as settlement substrate. Additionally, chicken egg shells were tested as potential substrate and were washed, then dried. All these shells (which are mainly composed of mineralized calcium carbonate) were glued onto Nunc 6-well multidishes (Fig S2b). Gastropod shells bearing *I. stephanieae* and bivalve shells bearing *I.* cf. *suecica* were placed into each well (supply shell with colony placed facing down onto the uncolonized substrate) filled with filtered seawater and placed in an incubator at 16 °C. These were supplied with 1 ml of diatom and microalgae cultures daily.

Experiment 1B: In addition, the two immergentiids (separate) were positioned with uncolonized shells mentioned above and *Littorina littorea* in baskets and left undisturbed, except when observed (Fig. S2c). These also received nutrition, as in Experiment 1A.

### Settlement experiment 2

Experiment 2A: For settlement, species of both *I.* cf. *suecica* and *I. stephanieae* were placed in food-enriched and non-enriched scenarios (Fig. S2f) in disks with a fine mesh.

Experiment 2B: Colonized (*I.* cf. *suecica*) and uncolonized molluscan shells were placed in a separate tank (shells placed in mini plastic shopping baskets (dimensions 15.2 D × 15.2 W × 7.6 H cm) and 50 ml falcon tubes tied with zip ties so baskets floated in the tank) and left undisturbed and fed with food cultures (Fig. S2 d and e). Shells were checked for larvae settlement every third day.

## Results

### Reproductive zooids

**Fig. 2:**
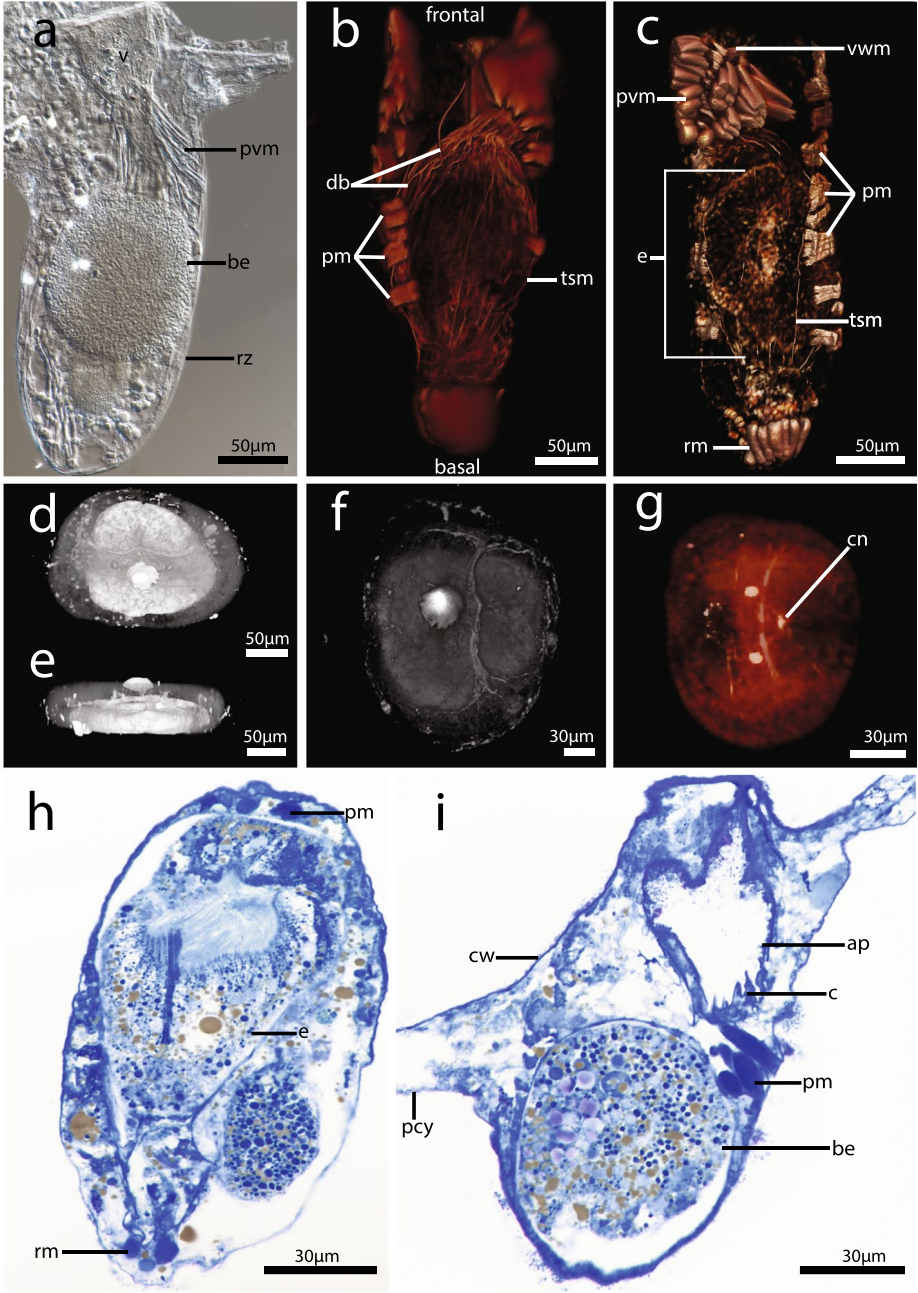
Reproductive zooids of *Immergentia*. **a** Whole mount of a reproductive zooid with a brooded embryo. **b – g** Volume renderings based on confocal scans. **b** Prominent musculature of the tentacle sheath, parietal muscles and duplicature bands. **c** Vestibular wall muscles, embryo within the tentacle sheath and retractor muscles. **d** Lateral top view of embryo, tilted 180° **e** Side view of same embryo **f** Lateral top view of embryo **g** Lateral bottom view of same embryo, α-tubulin staining for neuronal and ciliary structures and phalloidin staining for f-actin. **h** Oblique tangential section of a brooding zooid with embryo. **i** Section of the same zooid showing lower part of the embryo. Abbreviations: ap – aperture, be – brooded, embryo, c – collar, cn – central nerve, cw – cystid wall, db – duplicature band, e – embryo, pcy – primary cystid appendage, pm – parietal muscles, pvm – parieto-vestibular muscle, rm – retractor muscle, rz – reproductive zooid, tsm – tentacle sheath muscle, vwm – vestibular wall muscle

Embryos develop from large macrolecithal oocytes subsequently moving from the zooidal coelom to the tentacle sheath of autozooids where the polypide has largely degenerated; these are referred to as reproductive zooids (Fig. 2). The embryo is circular to oval shaped (Fig. 2 a–g) filling a large part of the body cavity. The lophophore and digestive tract are degenerated but especially the apertural, parietal, tentacle sheath, and/or retractor musculature remain in reproductive zooids (Fig. 2 a–c). The apertural muscles facilitate the opening and closing of the orifice of the zooid (Fig. 2 b, c). Retractor muscle bands originate from the proximal end of the cystid wall (Fig. 2 c, and h). Transverse parietal muscles occur laterally on the cystid wall, along the length of the reproductive zooid (Fig. 2 b, c, h and i).

### Settlement experiments

In Settlement Experiment 1A (16 August–16 October); a total of seven ancestrulae of *Immergentia stephanieae* settled on *Lutraria lutraria* (3) and *Anomia ephippium* (4). No settlement of either immergentiid was observed on egg shells, and no ancestrulae of *Immergentia* cf. *suecica* were observed on any substrates. In Settlement Experiment 1B, four ancestrulae of *I. stephanieae* settled on *L. littorea* shells but no settlement of *I*. cf. *suecica* was observed. Where more than one ancestrula of *I. stephanieae* was observed, they often occurred in close proximity to each other (Fig. S4, Ancestrulae 3, 4, 6 and 7).

In Settlement Experiment 2A (27 February–22 March), two potential ancestrulae borehole apertures of *I. stephanieae* were identified in the non-enriched, but none in the food-enriched setups*.* Two *I.* cf. *suecica* ancestrulae settled on a shell already containing colonies, none on uncolonized shells. In Settlement Experiment 2B, a total of 15 *I.* cf. *suecica* ancestrulae settled on molluscan shells over the duration of observations*.*

### Ancestrulae, colony development and morphology

**Fig. 3:**
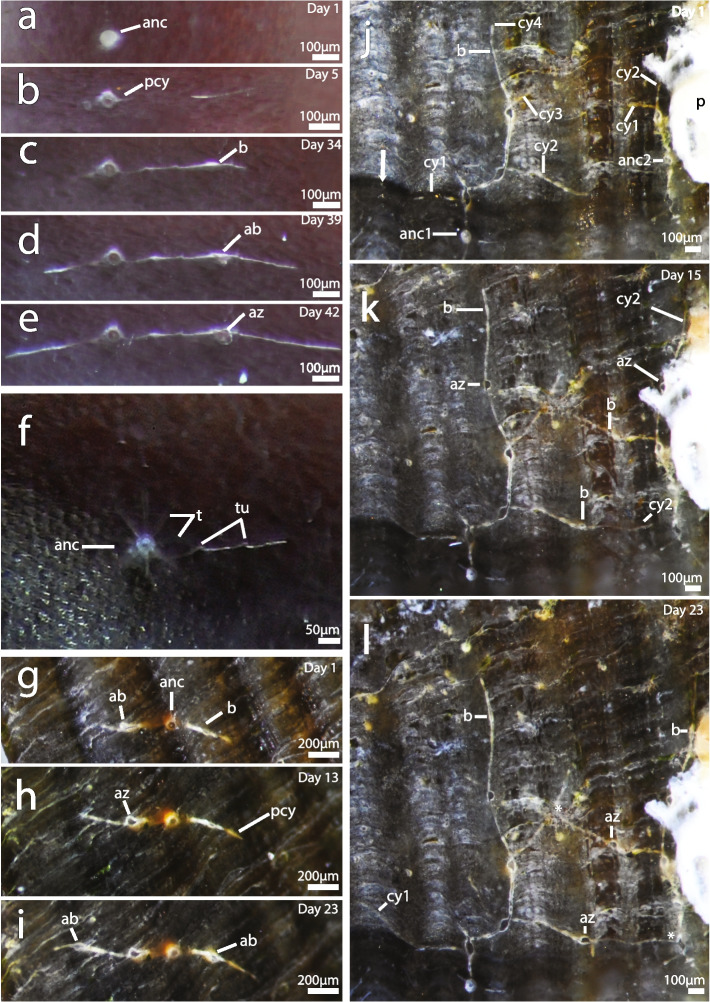
Small colonies of *Immergentia stephanieae* in Experiment 1*.* **a – e** Development of young colony from ancestrula over 37 days. **a** Day 1: Early development of ancestrula after settlement of larva on *Littorina littorea* substrate. **b** Day 5: Aperture break through and first primary cystid appendage (right) apparent. **c** Day 34: First bud and opposite (left) primary cystid appendage. **d** Day 39: Bud in advanced state (lateral expansion of bud). **e** Day 42: Bud break through surface of substrate into a feeding zooid. **f** Tentacle crown of ancestrula with eight tentacles (one tentacle obscured from view). **g** &** h** Young colony. **g** Ancestrula with buds (one advanced and one developing) on opposite cystid appendages. **h** Advanced bud break through substrate and expansion of primary cystid appendage. **i** Borehole apertures of inaugural autozooid and advanced bud deflected in opposite directions (enantiomorphism). Advanced bud (borehole not completely open) deflected in direction opposite to preceding autozooids. **j – l** Two small colonies growing toward each other. **j** Borehole aperture of one ancestrula (anc1) covered by glossy varnish (arrow) of gastropod. Second ancestrula (anc2) growing along polychaete tube. **k** Expansion of cystid appendage (cy2) toward second colony and bud developed. **l** Breakthrough of buds into autozooids and development of new buds. Abbreviations: ab – advanced bud, anc – ancestrula, az – autozooid, b – bud, cy – cystid appendage, p – polychaete, pcy – primary cystid appendage, t – tentacle, tu – tubulets

**Fig. 4:**
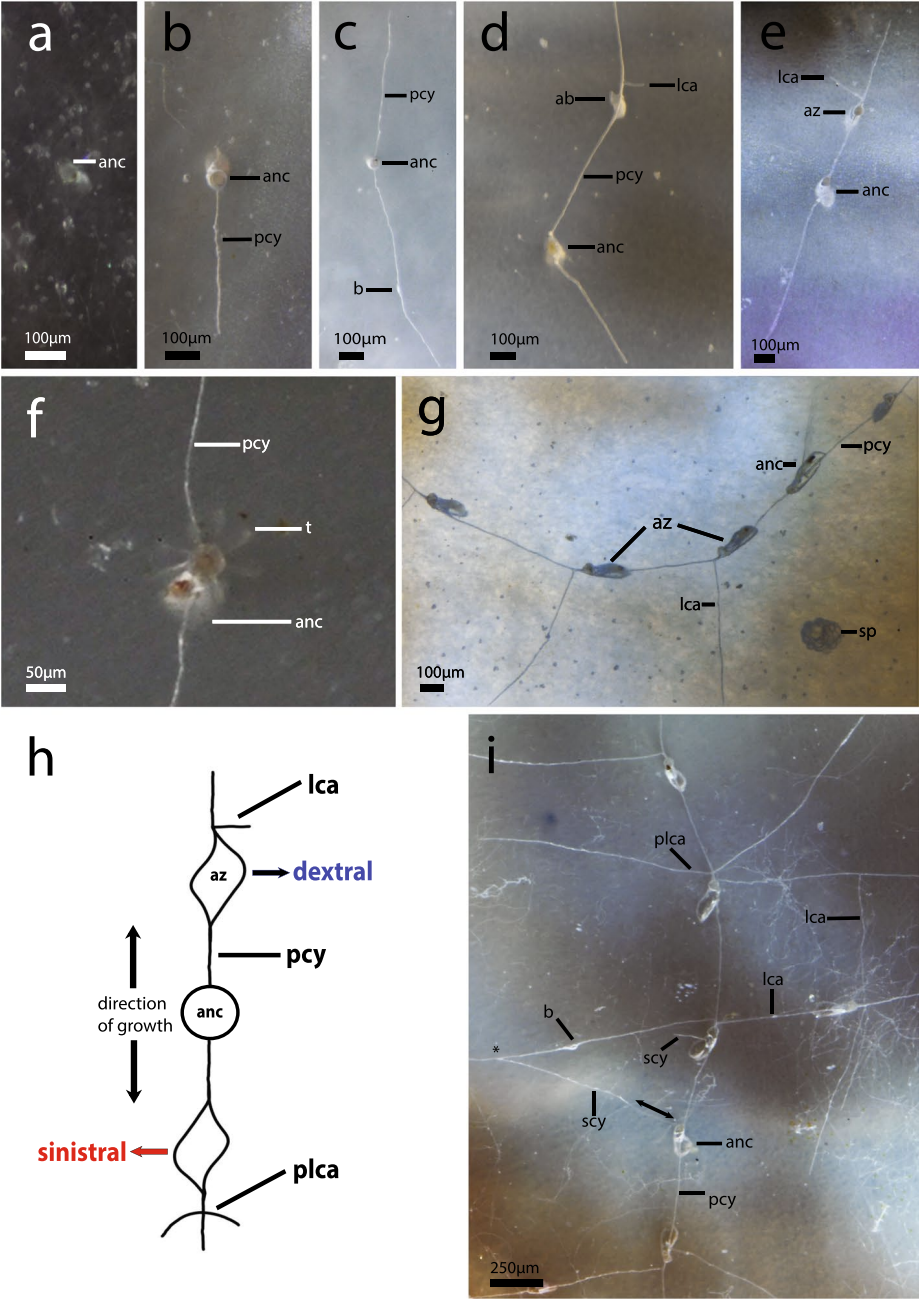
Small colonies of *I* cf. *suecica*. **a** Young ancestrula. **b** Ancestrula with one long primary cystid appendage, development of next primary cystid appendage (opposite direction) delayed. **c** Ancestrula with two primary cystid appendages. **d** Ancestrula with primary cystid appendages developing at an acute angle with developing bud in an advanced state (top), no breakthrough of borehole aperture yet. Lateral cystid appendage developing to the right of primary cystid appendage. **e** Ancestrula and autozooid after breakthrough of borehole aperture. Lateral cystid appendage developing to the left from primary cystid appendage near autozooid. **f** Tentacle crown of ancestrula with eight tentacles (one tentacle obscured from view). **g** Autozooids do not display enantiomorphism; all appear on the same side of the primary cystid appendage. **h** Schematic of sinistral and dextral borehole aperture deflection along the primary cystid appendage. Arrow indicates direction of growth from the ancestrula. **i** Same colony. Lateral cystid appendages develop only after development of first autozooids. Secondary cystid appendage developing from ancestrula below substrate surface (double arrowheads) then visible just below surface with tubulets and anastomose with lateral primary cystid appendage (asterisk) with developing bud. Lateral and paired lateral cystid appendages in section of colony. Autozooids neighbouring ancestrula are enantiomorphic. Abbreviations: ab – advanced bud, anc – ancestrula, az – autozooid, b – bud, lca – lateral cystid appendage, pcy – primary cystid appendage, plca – paired lateral cystid appendage, scy – secondary cystid appendage, sp – boring sponge, t – tentacle

The first indication of an ancestrula was a circular structure that developed into a borehole aperture with average sizes of 40.6 µm ± SD 4 (*n* = 11) in *I. stephanieae* and 38.2 µm ± SD 9 (*n* = 11) in *I.* cf. *suecica*. A small colony of *Immergentia* sp. from Japan had a borehole aperture of 40.9 µm (Table S2, Fig. S5 f). The borehole apertures of ancestrulae were distinguishable from those of autozooids (which are typically oval to spindle-shaped) in being, sometimes circular (depending on the species) and slightly larger in size (Figs. 3 and 4). The ancestrula borehole typically displays no enantiomorphism and in its initial stages is only distinguishable (from circular borings of other organisms) when a cystid appendage develops. Enantiomorphism refers to the mirror image relations, with borehole apertures of autozooids being either dextral (deflected to the right) or sinistral (deflected to the left) of the primary cystid appendage (Fig. 4 h). Lateral expansion of the primary cystid appendage developed before breakthrough of the autozooid. The duration of development of ancestrula into a feeding autozooid was between 10 and 14 days in both *I. stephanieae* and *I.* cf. *suecica*. Similarly, the ancestrula had eight tentacles in both species (Figs. 3f and 4f). The first bud developed only after maturity of the feeding ancestrula.

The first indication of autozooid formation is a bud (in the form of a swelling) derived from the cystid wall of the primary cystid appendage and engraved on the shell surface (Figs. 3c,k 7a, S3b). The primary cystid appendage may continue to develop further beyond the etching. Simultaneously, the borehole is dissolved by chemical means, downwards (basally) into the substrate. During this phase the future borehole aperture remains sealed until it breaks through the substrate surface and feeding can commence. Autozooid development lasted an average of 12 days ± SD 2.6 (*n* = 3) and from bud to zooid 11.7 days ± SD 3.5 (*n* = 3) (Table 1) in *I. stephanieae*. In a colony of *I.* cf. *suecica*, the bud development to a functional zooid occurred after seven days (under a food enrichment treatment).

**Table 1:** Growth rates of *Immergentia stephanieae*. State of ancestrula and colony size before and after observations for Experiments 1 & 2

		**Colony at start**					**Growth**			**Colony at end**					**Observations**	
**Experiment 1** **(16.08—16. 10, 2021)**	**Ancestrula**	**Young ancestrula** **(no breakthrough)**	**Feeding ancestrula**	**Number of cya (s)/growing edges**	**No. of auto-zooids**	**No. of buds**	**Total cya growth (µm)**	**Days observed**	**Growth rate day**^**−1**^ **(µm)**	**New cya (s) added**	**New zooids added**	**No. of buds into zooids**	**Advanced buds**	**New buds**	**Notes on new buds and zooids**	**Comments on substrate**
	1	-	✓	2	-	1	**560,2**	23	**24,4**	-	-	1	2	-	Boreholes of buds not completely open	*Littorina littorea* shell
	2	✓	-	-	-	-	**1224,5**	42	**29,2**	2	1	-	-	-	Both ancestrula and zooid feeding	From settlement experiment 1B. *Littorina littorea* shell
	3	✓		-	-	-	**133,3**	20	**6,7**	1	-	-	-	-	Extension of cya(s)	*Littorina littorea* shell
	4		✓	3	2	1	**671,0**	24	**28,0**	-	-	1	-	1	Bud into zooid = 15 days	*Littorina littorea* shell
	5		✓	1	-	1	**755,4**	21	**36,0**	-	-	-	1	-	Bud almost break through	*Littorina littorea*
	6		dead	4	3	1	**2315,7**	23	**100,7**	1	1	1	-	1	Possible fusion with ancestrula 7 New zooid = 14 days Bud to zooid = 12 days	Ancestrula sealed by gastropod varnish. *Littorina littorea* shell
	7		✓	2	2	1	**611,2**	23	**26,6**	-	1	1	-	1	Obscured part of cya extension (≈ 300 µm) omitted. Possible fusion with ancestrula 6 New zooid = 13 days Bud to zooid = 8 days	*Littorina littorea* shell
	8		✓	1	-	-	**10,8**	10	**1,1**	-	-	-	-	-	Extension of cya then growth seized	From settlement experiment 1A. *Lutraria* *lutraria* shell
	9		✓	1	-	-	**82,4**	8	**10,3**	-	-	-	-	-	-	From settlement experiment 1A. Shell *Lutraria lutraria*
	10		✓	3	1	2	**960,6**	19	**50,6**	1	1	2	-	-	Advanced buds to breakthrough 3 days New zooid = 9 days	*Littorina littorea* shell
							Mean = **732,5 ± SD 679**		Mean = **31,3 ± SD 28**							
**Experiment 2** **(27.02—22.03, 2023)**	T1		✓	4	3	1	**8,2**	22	**0,4**	-	-	-	-	-	Slight extension of cya(s) then halted	No additional nutrition. Ancestrula fed throughout experiment, autozooids to a lesser degree
	T1-F		✓	4	3	1	**504,9**	22	**23,0**	2	-	1	-	-	Extension of cya(s)	Added nutrition. All zooids actively fed

An autozooid tilts in the direction of deflection of the borehole aperture. In *I. stephanieae*, where enantiomorphism is obvious, the first zooid on one side of the ancestrula may display either dextral or sinistral deflection along the primary cystid appendage, and the zooid on the opposite side is deflected in the opposite direction (Figs. 3g–l). Similarly, the successive borehole apertures and zooids display either dextral or sinistral deflection depending on the preceding zooid. In *I.* cf. *suecica* this pattern is not consistent. Autozooids on either side of the ancestrula can all be deflected in the same direction and/or sometimes show enantiomorphism (Figs. 4g and i). The borehole aperture of the ancestrula is circular in *I. stephanieae* and *I.* cf. *suecica*, and does not display enantiomorphism (Figs. 3 and 4). However, in some instances the ancestrular zooid may tilt in one direction (Figs. 4e).

**Fig. 5:**
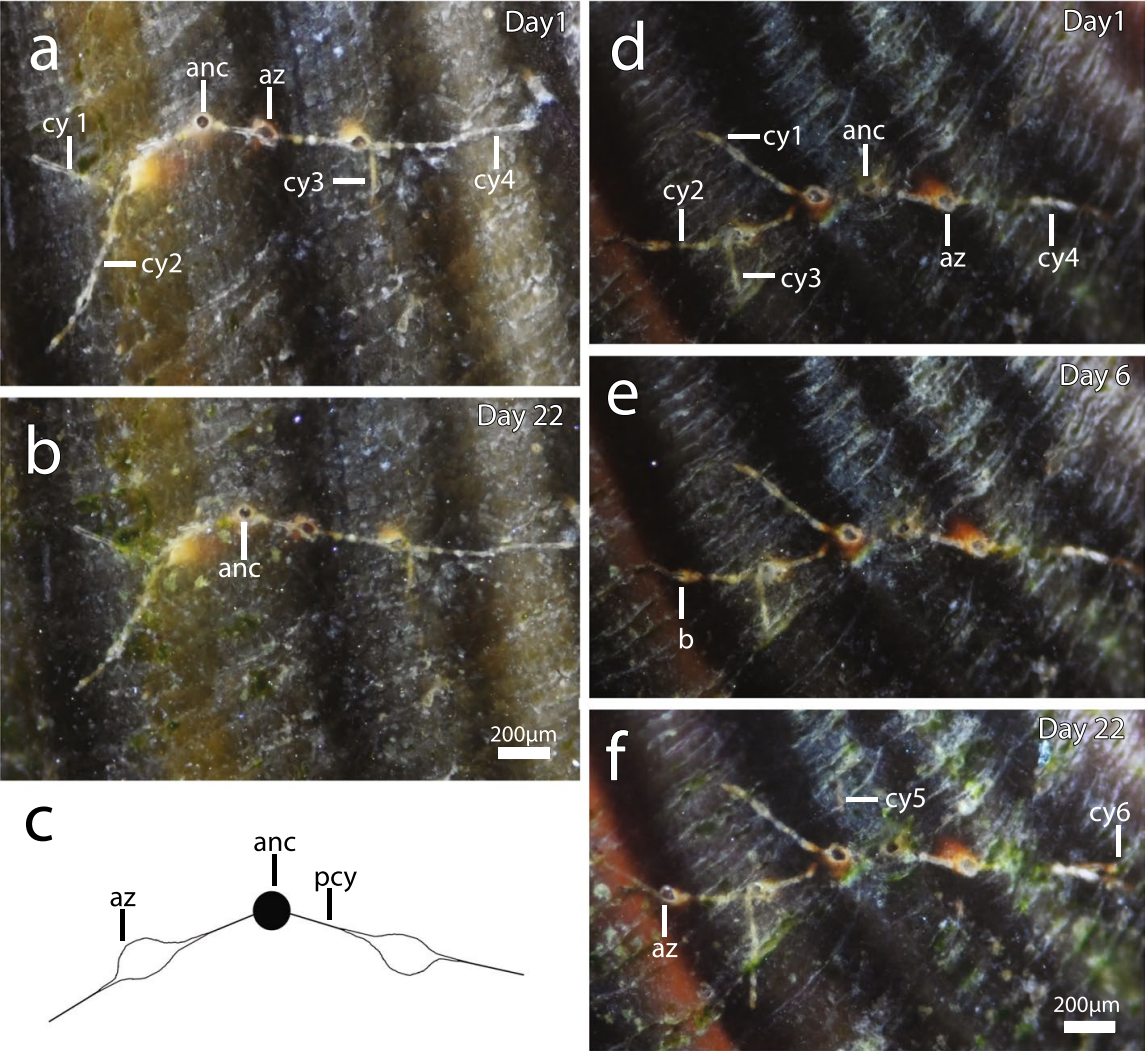
Early growth of *Immergentia stephanieae*. **a** Bryozoan colony GI1 at the start of the experiment under control condition. Two autozooids on either side of the ancestrula. Four cystid appendages monitored. **b** Same colony after 22 days. **c** General structure of colony and autozooids positioned opposite the ancestrula at an acute angle. **d** Bryozoan colony GI1-F under the food treatment at the start of the experiment. Four cystid appendages observed and two autozooids on either side of the ancestrula. **e** Same colony. Development of a bud on the second cystid appendage. **f** Bud developed into a feeding autozooid on the second cystid appendage after 22 days. Two additional cystid appendages developed, cy5 below the surface of the shell and cy6. Extension of cy3. Abbreviations: anc – ancestrula, az – autozooid, cy – cystid appendage, pcy – primary cystid appendage

Primary cystid appendage formation in ancestrulae occurs below the frontal apertural area or in the mid-zooidal region. It commences as a single primary cystid appendage followed by a delayed primary cystid appendage in the opposite direction (in the same plane). A secondary cystid appendage developed from an ancestrula where growth of the primary cystid appendage slowed/ceased or in other instances potentially served as an additional route. Lateral cystid appendages emanate from the primary cystid appendage, usually close to the apertures of an autozooid, or sometimes derived from the secondary cystid appendage (Figs. 4d, e,g and i; Fig. S3). Lateral cystid appendages may appear (distal of the aperture) opposite or alternate along the primary cystid appendage at regular or irregular intervals. In *I.* cf. *suecica*, paired lateral extensions are common along the primary cystid appendage and can form a feather-like arrangement (Figs. 4i and S3a). Sometimes lateral cystid appendages are not directly opposite each other but rather offset by a short distance (Fig. 4i). In *I. stephanieae*, the aforementioned paired pattern is not typical and a single lateral cystid appendage generally extends from the primary cystid appendage (Figs. 5a and d). Lateral cystid appendages are separated from other cystid appendages in the colony by septa and sometimes intercalary kenozooids (a kenozooidal tube derived from the cystid wall and inserts between neighbouring cystid appendages separated by a septum on either side; Fig. S3 b, c). Secondary cystid appendages can anastomose with the primary or lateral cystid appendages of other zooids, forming grid-like patterns in both species (Figs. 4i). Autozooids can develop on lateral cystid appendages. Sac zooids occur randomly throughout the colony and typically develop on lateral or secondary cystid appendages. Sac zooids are kenozooids filled with granules, lacking a distinct aperture.

**Fig. 6:**
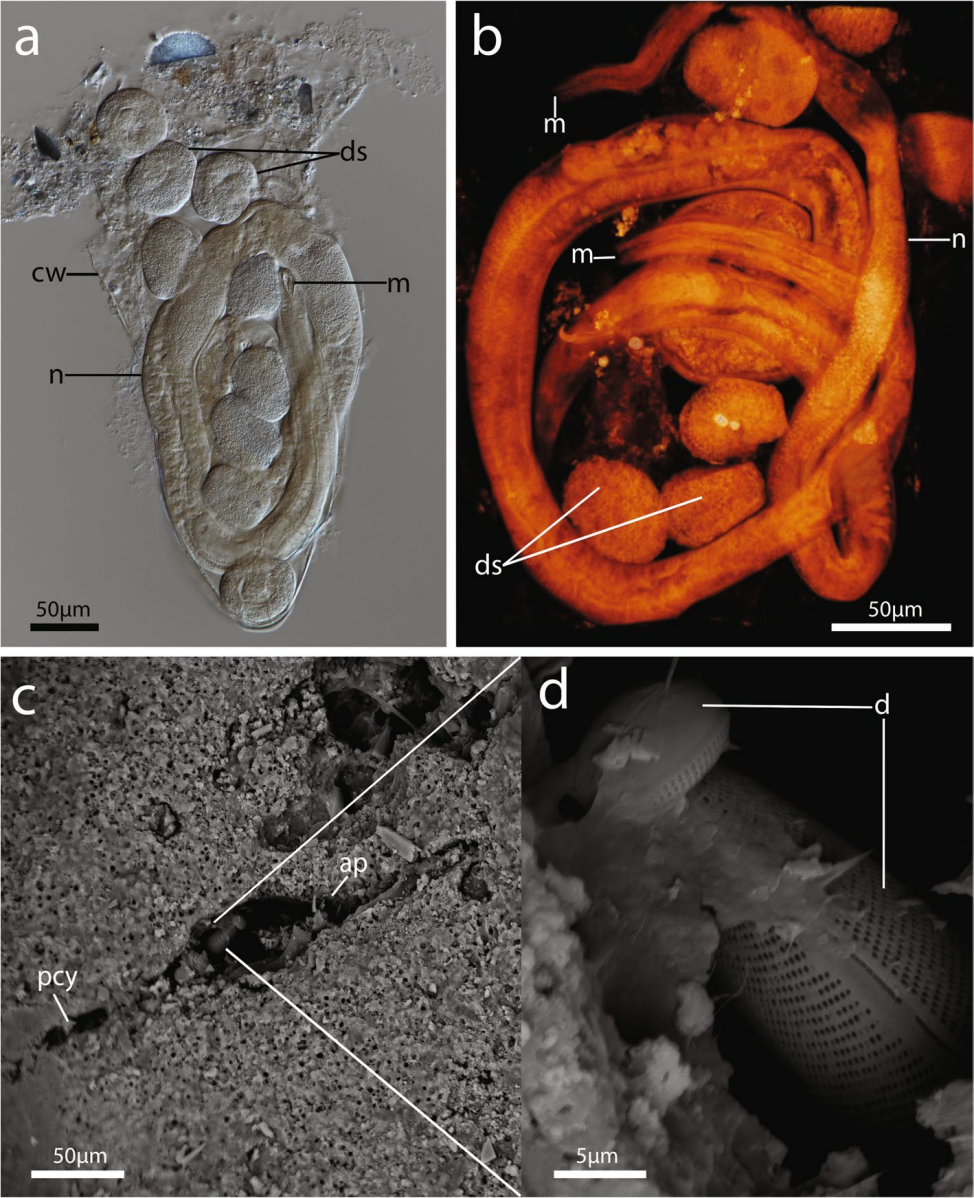
Cavities of immergentiids occupied by other organisms. **a** Whole mount of nematode in remaining cystid wall of *Immergentia patagoniana*. Developing early developmental stages visible. **b** Confocal laser scan of two nematodes as well as different stages of development in a cystid wall of *Immergentia patagoniana*. **c–d** Scanning Electron Microscope images of immergentiid borehole aperture. **c** Empty spindle-shaped borehole aperture of *Immergentia stephanieae* with diatoms. **d** Close-up of diatoms in borehole aperture. Abbreviations: ap – aperture, cw – cystid wall, d – diatom, ds – developmental stages, m – mouth, n – nematode, pcy – primary cystid appendage

Other organisms such as nematodes and their developmental stages occupied boreholes where the cystid wall was still intact in the subtidal species *I.* cf. *zelandica* (New Zealand) and *I. patagoniana* (Burdwood Bank) (Figs. 6a and b). Ciliates and diatoms were found residing in vacant immergentiid boreholes (Figs. 6c and d).

## Growth rates

### Growth experiment 1: growth rates of *I. stephanieae*

Growth was measured in a total of 10 small colonies of *I. stephanieae*, each initially consisting of an ancestrula and fewer than four autozooids. Seven of these 10 ancestrulae were found on the same *Littorina* shell while the others were from the settlement experiments (Table 1, Fig. S4). The growth rates varied among colonies with Ancestrulae 8, 3 and 9 having the lowest growth rates, of 1.1, 6.7, and 10.3 µm day^−1^, respectively. The highest growth rates of 100.7, 50.6, and 36.0 µm day^−1^ were observed in Ancestrulae 6, 10 and 5, respectively.

No growth was observed in dredged *I.* cf. *suecica* where three ancestrulae with a single cystid appendage (cya) and one small colony at the three zooid-stage were observed in addition to one shell with several ancestrulae (more than 10 single ancestrulae with 1 or 2 cya/s). Minimum length of observation was three weeks.

### Growth experiment 2: growth rates of *I. stephanieae* and* I.* cf.* suecica*

**Fig. 7:**
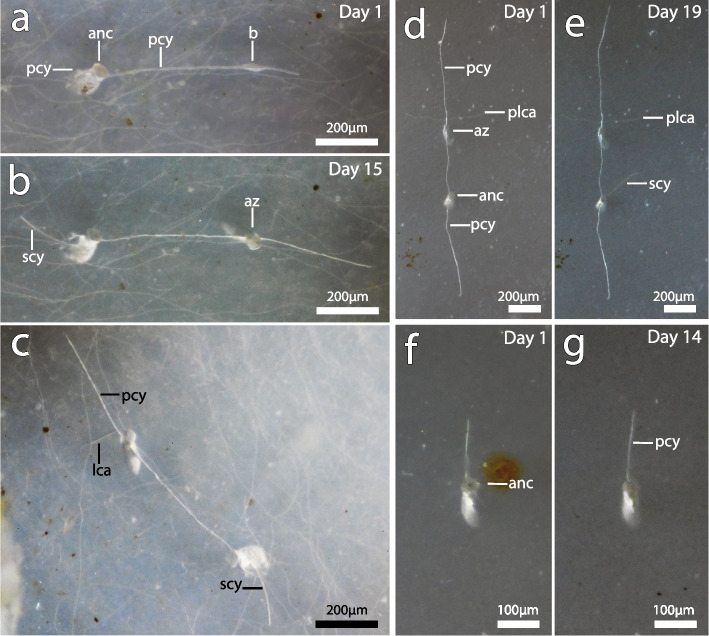
Early growth of *Immergentia* cf. *suecica* under the food enrichment treatment. **a–c** Ancestrula development over 15 days. **a** Ancestrula with bud developing on primary cystid appendage at start of the experiment. **b** Same colony with feeding autozooid after 22 days under the feeding treatment. Development of primary cystid appendage (halted/delayed). **c** Same colony viewed from different angle to show extension of lateral, primary and secondary cystid appendages. **d** Ancestrula and autozooid at start of experiment. **e** Growth of primary cystid appendage (top) and secondary cystid appendage developing from the ancestrula. Primary cystid appendage (bottom) did not grow. **f** Ancestrula with a single cystid appendage and tentacles protruded. **g** Extension of primary cystid appendage. Abbreviations: anc – ancestrula, az – autozooid, b – bud, lca – lateral cystid appendage, pcy – primary cystid appendage, plca – paired lateral cystid appendage, scy – secondary cystid appendage

Two small colonies of *I. stephanieae* of similar size were obtained and examined (Fig. 5). A total of 18 ancestrulae of *I.* cf. *suecica* were obtained (often several on the same shell), 15 of which consisted only of the ancestrula, two at the two-zooid-stage and one at the four zooid-stage. Only three of these colonies exhibited growth in the form of cystid appendage extension and/or bud/zooid formation (Table 2) in the food enriched treatment (Fig. 7).

**Table 2:** Growth rates of *I.* cf*. suecica*. Status of ancestrula and size of colonies at the start and end of observations for Experiment 2

		**Colony start**				**Growth**			**Colony end**					**Observations**		
**Experiment 2** **(27.02—22.03, 2023)**	**Ancestrula**	**Feeding ancestrula**	**Number of cya (s)/growing edges**	**No. of auto-zooids**	**No. of buds**	**Total cya growth (µm)**	**Days observed**	**Growth rate day**^**−1**^ **(µm)**	**New cya (s) added**	**New zooids added**	**No. of buds into zooids**	**Advanced buds**	**New buds**	**Notes on new buds and zooids**	**Comments on substrate**	
	S2-F	✓	4	1	-	**452,5**	20	**8,6**	1	-	-	-	-	Secondary cystid appendage developed from ancestrula when growth halted at one end of the primary cystid appendage	Additional nutrition. Ancestrula fed throughout experiment. Growth on *Ensis* sp. shell	
	S3-F	✓	1	-	-	**38,0**	14	**2,7**	-	-	-	-	-	Not obvious if second cystid appendage is present	Additional nutrition. Probing of tentacles observed. Growth on *Ensis* sp. shell	
	S4-F	✓	2	-	1	**593,8**	16	**16,5**	1	-	1	-	-	Established bud to autozooid = 7 days	Additional nutrition. Ancestrula fed throughout experiment. Growth on *Ensis* sp. shell	
						Mean = **361,4 ± SD 296**		Mean = **9,3 ± SD 7**

In the small colonies of comparable size TL1 (control, no food) and TL1-F (food enriched), the growth rate was 0.4 and 23.0 µm day ^−1^, respectively (Table 1). In addition to cystid appendage extension the TL1-F sample also grew an autozooid that was feeding by the end of the experiment. For larger colonies of comparable size, the control colony TL2 grew at 36.81 µm day ^−1^ while the colony that received the food-enriched culture grew less at 0.1 µm day ^−1^ (Table 3). In large colonies, TL3 and TL3-F growth in the control was slightly lower at 2.6 and 3.0 µm day ^−1^, respectively. Similarly, growth was higher in colonies that received food RS2-F at 1.9 µm day ^−1^ compared to the control RS1 with a growth rate of 0.4 µm day ^−1^. Interestingly, the large colony RS1, did not receive food but displayed the highest growth rate in this experiment at 74.9 µm day ^−1^.

**Table 3:** Growth rates of *Immergentia stephanieae* in nutrition and no-nutrition treatments in Experiment 2

				**Cystid appendage (cya) growth**				**Growth**		
**Colony size**	**No. of colonies observed**	**Sample**	**Feeding treatment**	**cya 1**	**cya 2**	**cya 3**	**cya 4**	**Total cya growth (µm)**	**Days observed**	**Growth rate day**^**−1**^ **(µm)**
**Small (ancestrula + 3 autozooids)**	**2**									
		TL1	x	4,16	1,98	2,02	-	**8,2**	22	**0,4**
		TL1-F	✓	406,64	4,27	398,89	-	**504,9**	22	**23,0**
**Colony size**	**No. of colonies observed**									
**Large > 30 zooids**	**4**									
		TL-2	x	406,64	4,27	398,89	-	**809,8**	22	**36,8**
		TL3	x	1,35	50,91	-	-	**52,3**	20	**2,6**
		TL4	x	3,25	0,92	-	-	**4,2**	20	**0,2**
		RS1	x	2,33	0,73	-	-	**3,1**	7	**0,4**
								**Mean = 217,3 ± SD 396**		**Mean = 10 ± SD 18**
**Colony size**	**No. of colonies observed**									
**Large > 30 zooids**	**5**	TL2-F	✓	0,28	1,43	-	-	**1,7**	20	**0,1**
		TL3-F	✓	40,99	18,75	-	-	**59,7**	20	**3,0**
		TL4-F	✓	26,75	232,54	-	-	**257,3**	20	**13,0**
		RS1-F	✓	310,17	380,03	247,35	561,09	**1498,6**	20	**74,9**
		RS2-F	✓	4,28	9,17	-	-	**13,4**	7	**1,9**
								**Mean = 366,6 ± SD 641,3**		**Mean = 23 ± SD 32**

In the food enriched samples the growth rates of *I.* cf. *suecica* varied at 8.6, 2.7, and 16.5 µm day ^−1^ for S2-F, S3-F and S4-F, respectively (Table 2).

### Seasonality implications

**Fig. 8:**
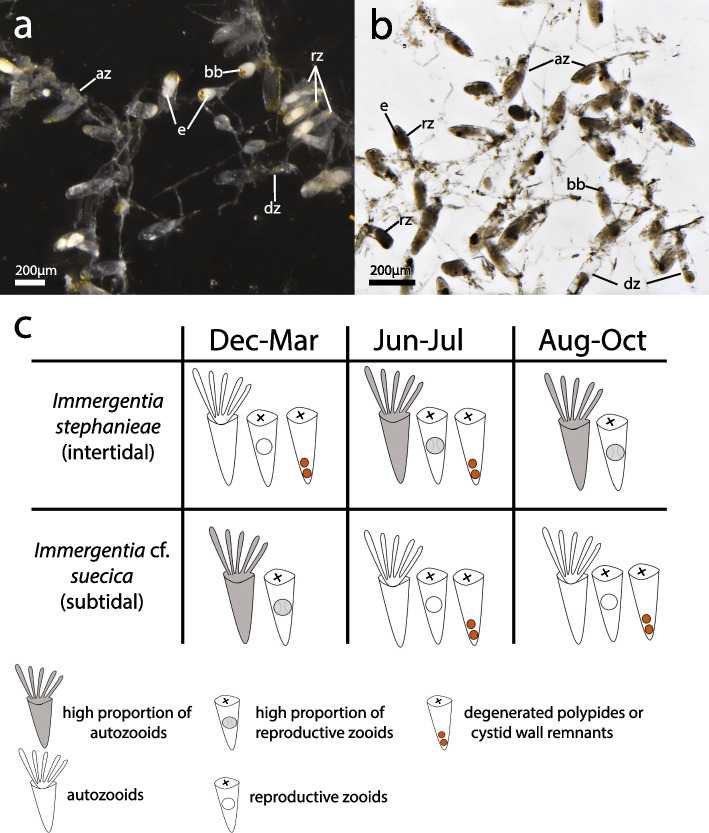
Proportion of zooids in colony over time. **a** Decalcified colony of *Immergentia stephanieae* during the peak reproductive period August to October. **b** Decalcified colony of *Immergentia* cf. *suecica* during the peak reproductive period of December to March. **c** Schematic representation of zooid proportions. During December–March, a single zooidal state does not dominate in *Immergentia stephanieae* colonies but in *Immergentia* cf. *suecica* autozooids and reproductive zooids are relatively abundant in the colonies. From June to July, there are relatively more autozooids and reproductive zooids in *Immergentia stephanieae* than in the period from December to March, while these zooids are more abundant during the peak period from August to October. For *Immergentia* cf. *suecica* a single zooidal state does not dominate from June to July and there are relatively even fewer autozooids and reproductive zooids from August to October but more zooids with degenerating and degenerated polypides or remnants of cystid walls. Abbreviations: az – autozooid, bb – brown body, dz – degenerated zooid, e – embryo, rz – reproductive zooid

Examinations of colonies collected at different times of the year indicate that there may be times when in *I. stephanieae* and* I*. cf. *suecica* the relative abundance of reproductive zooids in the colonies outnumbers that of zooids in other states, i.e., autozooids, degenerated polypides, or only cystid wall remaining (Fig. 8). From December to February *I*. cf. *suecica* colonies had more auto- and reproductive zooids in the colony, while in *I. stephanieae* neither auto- nor reproductive zooids dominated and colonies had zooids with degenerated polypides. In the period from June to July, *I*. cf. *suecica* had fewer reproductive zooids than in the peak period, while *I. stephanieae* colonies had more auto- and reproductive zooids. From August to October *I. stephanieae* colonies are inundated with a greater proportion of autozooids and reproductive zooids. On the other hand, *I*. cf. *suecica* colonies have fewer autozooids, a high proportion of zooids with degenerating and degenerated polypides and few reproductive zooids during the same period.

### Feeding behaviors and defecation

The following account is based on observations of *I. stephanieae* (video S1). When a large or undesirable object was present, the tentacles transferred it from one individual to another or the lophophore retracted quickly and the current created by this action whisked the particle away from the borehole aperture of the polypide or through the tentacle spaces. Flicking, and curling/caging of the tentacles, was observed (video S1). The lateral view of *I. stephanieae* (in a broken *Littorina littorea* shell) shows that there is some flexibility with the body wall showing contraction capabilities during polypide eversion (video 1). The cystid wall of an autozooid is not completely attached to the borehole (dissolved cavity in the substrate). When the lophophore is protruded, an indentation in the body wall (pulling away from the borehole) is observed. When the lophophore was retracted (muscles relaxed), the cystid wall expanded and the borehole was filled.

During the defecation process (video 1), the protrusion of the polypide moved the anus toward the surface of the borehole aperture (in the substrate). The faecal pellet (light brown color) moved from the intestine towards the anus and once the lophophore protruded the tentacles shifted to the oral side and the faecal pellet was expelled. Based on observations, tilting of the lophophore did not always occur, sometimes it remained upright during faecal expulsion. Autozooids probed and some zooids retracted the lophophore if faecal expulsion occurred in a nearby zooid and sub-lophophoral currents moved faecal pellets from the substrate surface.

## Distribution

Shells with immergentiids and immergentiid traces were obtained from known and newly reported locations (Fig. 1). An immergentiid from Helgoland in the North Sea (Fig. S5 a, b) is confirmed. Immergentiid traces are reported for the first time from Pago Bay and Family Beach in Guam (Fig. S5 c–e); Sagami Bay and the Enshu Sea in Japan (Fig. S5 f, g) in the Pacific Ocean, and the Caribbean Sea in Guadeloupe; between the south of Basse-Terre and Marie-Galante (Table S1, S2, S5 h). In addition, borehole apertures belonging to the genus were found in the molluscan shell collection from the Senckenberg am Meer and for the first time reported from Tenerife in the Canary Islands, the Moroccan coast and Sicily, Italy. Additional samples were from the Norwegian Sea at a depth of 119 m, the Bay of Biscay Penmarch, France at a depth of 610 m and from Enshu Sea in Japan at a depth range of 153 to 180 m. The French sample represents the greatest depth of collection reported for immergentiids thus far. Although samples from the various polar regions (Arctic, Antarctic) were examined, no traces of boring bryozoans were found. Similarly, gastropods were collected from the intertidal zone of Île de Batz a small island near Roscoff, France, but no evidence of immergentiids or other boring bryozoans was found. The inventory of shells bored by immergentiids is expanded to include gastropod species such as*, Crepidula fornicata*, *Tritia reticulata*, *Drupa morum*, and *Fusinus colus*, and bivalves such as *Polititapes aureus*, *Pseudamussium clavatum*, *Bursa thomae* and *Chlamys islandica* (Table S1).

## Discussion

### Reproductive zooids

There are differences in the prevalence of reproductive zooids in colonies throughout the year, in warmer (August to October) and colder months (December to March) for *I. stephanieae* and *I.* cf. *suecica*, respectively. However, reproduction probably occurs continually throughout the year owing to the regenerative capacity of the polypides of these bryozoans, as well as the presence of reproductive zooids in a few colonies (of both species), albeit to a lesser extent than in the periods mentioned above. Fertilization occurs internally in gymnolaemates [54, 55]. Immergentiids are internal brooders similar to other ctenostomes (see [7, 11, 56]) and during embryonic development the polypide degenerates [9, 23] with exception of the musculature used for larval release from the tentacle sheath through the orifice. The vestibulum is also longer in reproductive zooids compared to autozooids, perhaps to aid larvae release [23].

### The founding zooid

In all bryozoans, metamorphosis occurs in two phases. First, the larval morphology is rearranged to form the pre-ancestrula, which then develops into a mature zooid with a polypide. The duration of the first phase is rapid, lasting a few minutes while the second phase can last for several days [57, 58]. Although no larvae were observed in our experiments, their presence was evidenced by the development of several ancestrulae on previously uncolonized substrates. The reproductive state of the ‘supply’ colonies (used for the settlement experiments) was unknown, i.e. whether fertilization had already occurred, developmental state of embryos and temporal-proximity to larval release. The duration of ancestrula development in both *I. stephanieae* and *I.* cf. *suecica* lasted between 10 and 14 days compared to 12 days in the boring bryozoan *Penetrantia clionoides* [59], which corresponds to the average autozooidal development of *I. stephanieae* of 12 days. It is challenging to draw inferences from these values because of the high variability in growth. The ancestrulae of *I.* cf. *suecica* and *I. stephanieae* had eight tentacles and were slightly smaller in size than the autozooids, which had nine and/or 10 tentacles, respectively. It is common for ancestrulae to be smaller than autozooids in bryozoans, as seen in cheilostomes [60–64] and cyclostomes [57, 65, 66].

Extant and fossil boring ctenostomes were classified based on the number and orientation of stolons and cystid appendages produced near or from the ancestrula (see [10], text Fig. 6, pp.142). Notably, immergentiids lack true stolons (kenozooidal polymorphs) but possess typical cystid appendages (extensions of the body wall), and also occasional intercalary kenozooids [9, 23, 50]. Two cystid appendages are typical for immergentiids and *Terebripora ramosa* [10], which is confirmed by observations in this study to have two primary cystid appendages typically extending in opposite directions from the ancestrula of immergentiids. However, both appendages do not always occur simultaneously.

In contrast to the ancestrula, the primary cystid appendages of autozooids typically develops in the frontal apertural area and secondary cystid appendages forms in the mid-zooidal region. Paired secondary cystid appendages can develop in autozooids at different angles [23]. The development of more than one secondary cystid appendage in ancestrulae is rare and likely occurs if the extension of the primary cystid appendage is hindered or halted. The secondary cystid appendage of one zooid can attach to the primary cystid appendage of another zooid (separated by a septum) or appear interposed with intercalary kenozooids [23]. Cystid anastomoses seem to be lacking in the early astogeny of immergentiids. This difference may be attributed to colony density, the age of zooids which can be indicated by the number of regeneration cycles within a colony [67] or the connectedness of zooids within the substrate i.e. enhanced communication as the colony expands.

When two cystid appendages are initially present they emanate in opposite directions from the ancestrula at the greatest possible angle to one another [10]. Deviations are exceptional, but can occur, e.g. in an unidentified immergentiid from the Pliocene (see [10], plate 23, Figs. 1– 3). In contrast, we also observed that opposite cystid appendages developing at oblique angles are common in *I. stephanieae* and *I.* cf. *suecica*. The influences of factors such as dissolution or curvature of the substrate are not known and difficult to determine. Nonetheless, multi-directional growth/budding possibly bears the advantage that the colony can continue to expand or redirect resources if unfavorable conditions are encountered on one end.

### Colony development

The borehole apertures of autozooids can be enantiomorphic, and therefore show either dextral or sinistral deflection along the primary cystid appendage [10]. Typical enantiomorphic boreholes are found in *I. patagoniana* and *I. stephanieae* while species with oval borehole apertures (e.g. *I. suecica*) display a lower degree of enantiomorphism and to an even lesser extent, or not at all, in those with circular borehole apertures, e.g. *I. zelandica* (see [10, 23]; S4 and S5).

Lateral cystid appendages only develop after the formation of an autozooid (at the growing edge) but not along the primary cystid appendage connecting the ancestrula and inaugural autozooids (this study). We postulate that the presence of lateral cystid appendages emanating from the primary cystid appendages of autozooids or the absence thereof may be species-specific. Paired lateral cystid appendages do not occur in the type species *Immergentia californica* [9], which however could not be confirmed (see [23]; Fig S1). Lateral cystid appendages can emanate from the cystid wall near the primary cystid appendages as also depicted in *I. californica* by Silén [9: Fig. 65, p. 43]. Adventitious cystid appendages occur sporadically throughout the colony and provide alternative routes for the exchange of materials between zooids should principal cystid appendages stop functioning [10]. Most immergentiids are capable of these interconnections, possibly improving distribution of nutrients within the colony (see [23]). In larger colonies it is challenging to distinguish primary from secondary cystid appendages, especially in non-translucent shells, a problem previously highlighted by Silén [9], which underlines the importance of observing early colony development.

### Growth rates in boring bryozoans

The growth rates of ancestrulae and already established immergentiid colonies are described here for the first time. These results were compared with data of penetrantiids [59], the only other study on growth rates of a boring bryozoan. However, these should be treated with caution as experimental set-ups, average seawater temperatures, and locations differed. Here, the average growth rates of *I. stephanieae* ancestrulae was 31.3 µm day^−1^ ± SD 27 was variable and higher than that of the penetrantiids colony *P. clionoides* with 28.8 µm day^−1^ (see [68]). Although both species are intertidal, the mean seawater temperature differed: a tropical environment at 28–30 °C for *P. clionoides* (see [59]) and temperate for *I. stephanieae* at 16–18 °C. For established colonies, it is even more challenging to draw comparisons. Although both *I.* cf. *suecica* and *Penetrantia* sp. are subtidal species from Roscoff, France, growth rates of the former were lower than the latter. Yet, for *I.* cf. *suecica*, average seawater temperature was 11 °C, with or without food-enrichment, and for *Penetrantia* sp. it was 16–18 °C (see [59]). In line with expectation, the growth of two large colonies of *P. clionoides* was greater than that of *Penetrantia* sp. [59] and *I.* cf. *suecica*. This study shows that growth rates between individual colonies are extremely variable and that the number of colonies studied is important for interpretations of growth rate. Similar to previous studies, temperature (see [13, 30, 36]) and nutrition [39, 68, 69] are important factors, as well as others which were not considered here such as colony age and size, phenotypic differences, carbonate chemistry, and feeding rates, all of which can also influence growth rates (see [32, 35, 39, 40, 70]).

### Growth rates under additional nourishment

Typically, food positively affects growth (see [33, 68, 69]) although results can differ under various factors such as salinity, temperature, flow velocity, pH (see [32, 39, 68]). The small colonies of *I. stephanieae* treated with food grew faster than those in the control. At the end of the experiment autozooids of both colonies were still feeding. However, for larger colonies there was no clear pattern of growth. Two of the large colonies in the control (TL2 and RS1) grew faster than those in the food-nourished group. This could be attributed to a number of factors. First, the colonies could be of different ages or growth by expansion and budding may have occurred at an area that was not observed. Due to the size of the colony (dense and large surface area), there could be more than one colony since the ancestrulae were not discernible in these samples. In the last sample, the only available space on the gastropod was its apertural region as the rest of the shell was overgrown.

### Challenges in measuring growth parameters

There are challenges associated with measuring and evaluating growth of boring bryozoans as the stolons, cystid appendages, buds, and zooids that formed all need to be considered. The last two structures require more energy/effort/time to develop. Determining growth has been studied in other bryozoans, especially encrusters with various growth forms (see [42, 71]), either by counting the number of zooids, the surface area or dry weight. Bryozoan growth rate measurements were recently reviewed by Smith and Key [43], but no guidelines exist for boring bryozoans. Similarly, studies used different parameters such as, growth rate [72–74], specific growth rate [30, 33], and growth efficiency [39, 70, 75] to indicate growth, sometimes with different units, posing a challenge for comparisons.

### Feeding behaviour

Feeding behaviour has been analysed in several recent studies [76–78], and was summarized by Winston & Migotto [44]. In immergentiids, the polypide can remain in a probing position, often only the collar and the tips of the tentacle poke through the aperture, before the tentacles are fully extended and the tentacle crown opened. The feeding behaviour corresponds to those observed in other bryozoans [see [34, 79–81]. Similar to other studies, the tentacles display a ‘disgusted attitude’ for undesirable conditions or particles ([54, 56*Conopeum seurati* did not feed when its food source was changed [29]. We observed that when a particle was too large or undesirable a series of retraction and eversion action was displayed, or quick retraction of the polypide. Sometimes the particle was enclosed in the tentacle crown in a curling motion of the tentacles inwards, then retracted or released through the gaps between neighbouring tentacles to dispose of it. Autozooids also transferred particles from one tentacle crown to another.

### Colony density, feeding and defecation

Colonies of *I. stephanieae* are more densely packed compared to those of *I.* cf. *suecica* [23]. There are advantages and disadvantages of short or long inter-zooidal spaces when related to feeding, defaecation, and flow velocities or water currents. Potential differences in faeces expulsion might result from different positions of the anus in ctenostomes [82]. In immergentiids the anus is located in the low or mid lophophoral region of the tentacle sheath [23]. Suspension feeding is enhanced in densely packed colonies [37, 83] but faecal expulsion from a lophophoral anus could interfere with feeding currents [34, 82]. Other species have adaptations, such as chimneys, for exhalant currents depleted of nutrients and faecal matter disposal [44, 77, 78, 84]. We observed that in *I. stephanieae* not all zooids protrude their lophophores at the same time during feeding, thus possibly regulating colonial feeding currents. A similar strategy was employed where interaction with the faecal matter occurred with closely spaced zooids. Increasing coordination outweighs the consequences of intracolonial competition [83]. The same strategy probably exists for substrates colonized by several intertwined colonies.

For species with large zooidal distances, such as *I.* cf. *suecica,* feeding currents may not be as efficient as in a densely packed colony (see [82, 85]). However, the advantage is that each zooid has a larger surface area from which to filter feed and the possibility of interference or interaction with the faecal pellets of neighbouring zooids is reduced. Four different pathways for faecal disposal in bryozoans, either by passing the faecal pellets through the lophophore (Pathway 1 & 2) or not (Pathway 3 & 4) [34]. Immergentiids display pathway 3, in which the faecal pellet is expelled through the anus and carried away by the sub-lophophoral current (upstream current).

### Inter- and intraspecific species interactions

#### Interactions of boring bryozoans

Self-fusion, merging within the same colony, is common in bryozoans (see [86–88]) while formation of anastomoses, merging of cystid appendages within the same colony, is common in arachnidiid bryozoans (see [7, 45]). In immergentiids this occurs when the secondary cystid appendage of a zooid connects to lateral or primary cystid appendages/intercalary kenozooids [23]. For the first time, we report the potential fusion of two small colonies of *I. stephanieae,* pending histological confirmation. Bryozoan fusion events may be manifest, thus physical and physiological connections require further examination [88]. Fusion has been studied in other sessile colonial organisms such as hydrozoans [89–91], corals [92–94], sponges [95, 96] extant cheilostomes [87, 88, 97, 98] and also fossil fenestrate bryozoans [99]. However, fusion may occur only in closely/genetically related individuals [86, 100–102]. Kin-recognition mechanisms are present in the larvae of *Bugula neritina* where siblings tended to settle close to one another [102]. Close settlement of several ancestrulae was observed in shells with either *I. stephanieae* or *I.* cf. *suecica* but their degree of relatedness, if any, is unknown. Alternatively, short-lived lecithotrophic coronate larvae (see [13]) may have limited dispersal capabilities or a large number of larvae may be produced, increasing the likelihood of close settlement. However, several colonial species reportedly possess self/non-self-capabilities thus permitting the recognition of compatible colonies [98, 101].

Different genera of boring bryozoans commonly occupy the same substrate with no clear indication of dominance, as stolons and/or cystid appendages typically overlap. We hypothesize that, the limitations are substrate condition, available space and colony age and their regenerative and growth capacity to survive. In addition, exploitation of different food resources and feeding currents (whether coordinated or not) could influence species interactions. The former is inferred on the basis that boring bryozoans have different modifications in the cardiac region of the digestive tract. Immergentiids have a cardiac constrictor [23], penetrantiids have a proventriculus [24] and spathiporids possess a gizzard ( [103], Johnson et al., in prep). Ingestion by bryozoans is influenced by particle size, phytoplankton species preferences and other factors (see [1, 44, 54]), and reports show that bryozoans with gizzards can crush diatoms [29, 41, 45, 104]. However, further research is required to confirm whether boring bryozoans have different diets.

#### Immergentiids and their substrates

Immergentiids have been reported on a variety of intertidal and subtidal molluscan substrates ([23, Table 2]), our data. Settlement experiments revealed that *I. stephanieae* larvae settled on shell fragments of *L. littorea* and bivalves collected from the subtidal zone. Therefore, their settlement is possibly attributed to the best available substrate in the habitat. Nevertheless, if colonies settled in the absence of their typical substrate, it would be interesting to determine whether there is substrate preference. In the intertidal zone *L. littorea* and *N. lupella* are among the larger gastropods (see [105, 106]) compared to other littorinid species, which were not colonized by immergentiids (see [23]), thus having the advantage of larger surface area for the colony to expand. The colony can expand as the gastropod grows or larvae settle on a large gastropod with sufficient space for expansion. Alternatively, the biomineral composition and effort required to dissolve mollusc shells (energetic cost) may influence immergentiid settlement. Analysis of shell compositions of *L. littorea* and *L. saxatilis* (among other gastropods) showed that on average proportions of organic components (versus mineral components) were significantly higher in the smaller compared to the larger shells, with comparably higher percentages reported for *L. saxatilis* (see [107]). The organic component of shells can increase its fracture toughness by up to three orders of magnitude [108, 109]. In addition, shell microstructure and minerology vary within Littorinidae [110].

Evidence, shows that older [111–114] and larger shells [112] or areas where the periostracum is thin or eroded [113, 115], tend to be heavily bored. The periostracum is the outermost protective proteinic layer of molluscan shells [116, 117] that can diminish over time as a result of wear or decay [10, 113, 118]. Boring bryozoans are still able to penetrate intact periostraca and calcareous layers [9, 112]. In other studies, older and larger shells were typically preferred and heavily bored [111, 114] or were species-dependent, as reported for endolithic cyanobacteria [119].

Unlike subtidal immergentiid species that typically occur in dead bivalves (see [23]), the intertidal *I. stephanieae* typically colonizes living intertidal gastropod species such as *L. littorea* and *N. lupella*. The advantages of boring bryozoans settling on living molluscs may generally be similar to those described for epibionts (see [120–122]). In the intertidal zone, gastropods can move to favourable areas, such as tide pools and below rocks/boulders during low tide, or find refuge between the fronds of macroalgae. We postulate that boring bryozoans have nutrients available when in tide pools and also protection from desiccation. Immergentiids typically occur in the apertural area of a living gastropod (especially the inner lip) where a varnish layer can seal autozooids. The varnish (shiny) layer or callus is secreted by a mantle associated with the foot epithelium [123, 124] and deposited on the parietal (inner lip) of the gastropod’s aperture but can extend onto the body whorl [124].

On the other hand, inactive substrate fragments (1) are prone to overgrowth with microalgae and other organisms in aquaria over time, a problem common in laboratory experiments, and (2) present limited space for colony expansion (if on small pieces) and (3) siltation. However, an advantage of inert substrates is that the protective periostracum is usually worn (see [9, 24]) therefore fostering settlement and expansion.

### Symbiosis, predation and other interactions

The boring bryozoans do not directly harm the host because they only occupy the shallow layers of shells (see [9, 10, 23, 53, 59]) without contact to the tissues as shown in living gastropods (see [53, 125]). This is indicative of a commensalistic relationship (one individual benefits whereas the other is neither aided nor harmed). However, this symbiotic relationship is multifaceted. For example, the boring sponge *Cliona* sp., did not directly harm *L. littorea* but heavily bored shells increased their vulnerability to predation by crabs and likely also affected their fitness [125]. If future research reveals that boring bryozoans have a similar effect, the status of the interaction would be considered parasitic (one individual benefits whereas the other is harmed) or mutualistic if both individuals benefit. There are several examples of commensalism [126, 127], fouling [128, 129] and mutualistic relationships [44, 130, 131] between bryozoans and other species (see [122]).

For the boring bryozoan, erosion and abrasion of the upper shell layer (essential for protecting the soft-body morphology) may lead to habitat loss and/or colony death, especially if deeper layers are unfavorable or unsuitable for settlement. We hypothesize that when the bryozoan colony dies (or the cavity becomes devoid of the opportunistic organisms that settle in it), the shell/affected part of the shell may be compromised and prone to degradation, formation of crevices, susceptible to dissolution and colonization as shown in other studies on coral and internal bioeroder relationships [132–136] especially in a more acidic ocean [137–142]. Boring sponges, polychaetes, and other epibionts that subsequently settle on a substrate with established boring bryozoan colonies appear to have a competitive advantage. The viability of such a colony would then depend on colony morphology (densely packed zooids vs large interzooidal spaces), reproductive state, substrate availability, and extent of substrate damage (see [10]). Possible responses of immergentiids include the diversion of growth direction or reallocation of resources (see [39, 40]) to enhance expansion.

General information on predators of bryozoans is well documented for pycnogonids [44, 143, 144] and nudibranchs [145–147] that feed on individual bryozoan zooids or entire colonies as well nematodes [148, 149], copepods, and gastropods (see [46, 150]). However, data on boring bryozoans is lacking. A boring lifestyle offers protection. Quick retraction of the lophophore is effective and only specialized predators can suck individual zooids out of the borehole. It is not uncommon for other organisms or ‘nestlers’ to occupy vacant boreholes. However, nematodes were found occupying remains of several *I. patagoniana* zooids and to a lesser extent of *I.* cf. *zelandica,* both of which are species from the subtidal zone. It is not clear whether the nematodes predate on the bryozoans while they are alive, opportunistically invade when zooids are vulnerable during degeneration or senescence or simply make use of the borehole where zooids died. The nematode *Oncholaimus dujardinii* was reported as a predator of non-boring bryozoans and fed on individual zooids [148]. Similarly, *Pelagonema obtusicauda* invaded colonies of *Electra pilosa* by entering via the pore plate [149]. Subsequently, the zooids were destroyed by this action and the nematodes used their cavities as housing. Perhaps the invasion occurred accidently [149]. The extent and frequency at which invasion of zooids occurs in living colonies of boring bryozoans are not known and require further investigations.

### Distribution

Despite the paucity of information, immergentiids have been reported in temperate and tropical seas. The presence of *Immergentia* in the North Sea was confirmed, but species identity remains unclear [151]. The greatest recorded depth of collection for immergentiids was 400 m (see [26]) but further details of the species and location were missing. Our record of 610 m from the Bay of Biscay Penmarch, France represents the deepest finding. There are several reports of fossil and extant immergentiid or immergentiid-like traces in grey literature (i.e. data outside traditional or commercial publishing), but many remain enigmatic [10], because it is challenging to assign species based on borehole apertures alone. Moreover, unpublished data, museum and personal collections especially of mollusc shells could expand our knowledge of their true distribution and diversity. Therefore, it is expected that the locations mentioned herein are not exhaustive.

The prevalence of immergentiids appears patchy. For example, immergentiids were ubiquitous in mollusc shells from Roscoff, France both in the intertidal and subtidal zone compared to penetrantiids, which were only found in the subtidal and not intertidal in Roscoff. On the contrary, penetrantiids dominated in the intertidal of Guam and only three gastropod shells bearing immergentiid borings were found (two intertidal, one subtidal). Perhaps their scarcity and dispersal can be attributed to a smaller population, water currents and physiochemical conditions as well as substrate properties such as shell buoyancy, shell size and substrate availability.

## Conclusions

The growth rates provided here can serve as a baseline for future growth estimates of immergentiids. The development of primary cystid appendages in opposite directions of the ancestrulae was similar in the species studied here. Therefore, early colony formation alone is not a robust diagnostic for distinguishing immergentiid species. but perhaps for the genus. Aspects regarding, the lifecycle as well as inter- and intraspecific interactions are still lacking. However, this work highlights these topics and presents different hypotheses to steer future research.

## Supplementary Information

Supplementary Material 1.

Supplementary Material 2: Video1- Immergentia stephanieae defecation figshare private link: https://figshare.com/s/b0a26c383b135c42fd9b.

## Data Availability

The data are available upon reasonable request.
