# Supplementary material for: Ecology of endolithic bryozoans: colony development, growth rates and interactions of species in the genus Immergentia
Source: Zoological Lett. 2024 Dec 31;10:23. doi: 10.1186/s40851-024-00246-9 (PMC11686985; doi:10.1186/s40851-024-00246-9)
Supplement: Supplementary file 1 — Supplementary Material 1. [file 40851_2024_246_MOESM1_ESM.pdf]

Supplementary Information

Ecology of endolithic bryozoans: colony development, growth rates and interactions of species in the genus *Immergentia*

Mildred J. Johnson[1$], Sarah Lemer[2], Masato Hirose[3], Sebastian H. Decker[1], Thomas Schwaha[1]

[1] University of Vienna, Dept. Evolutionary Biology, Djerassiplatz 1, A-1030 Vienna, Austria

[2]Marine Laboratory, UOG Station, Mangilao Guam 96923, USA

[3]School of Marine Biosciences, Kitasato University, Kitasato 1-15-1, Sagamihara-Minami, Kanagawa 252-0373, Japan

[$] corresponding author: mildred.johnson@univie.ac.at

ORCID:

MJJ: 0000-0003-0987-1374

SL: 0000-0003-0048-7296

MH: 0000-0001-9991-7238

SHD: 0000-0001-9029-8717

TS: 0000-0003-0526-6791

Table S1. Reports of recent immergentiids on substrate from literature and examined in this study.

Immergentia species	Class	Substrate	Substrate condition	Reference	Location (s)
angulata	*Gastropoda*	*Pisania tritonoides* (Reeve, 1846)	Possibly living and/or dead gastropods	Soule & Soule, 1969	Collected 15 July 1967, depth 45 Feet (13.7 m), water temperature 81 °F
californica	*Gastropoda*	*Acanthinucella spirata* (Blainville, 1832)	dead	Soule, 1950	California, Cabrillo Beach, San Pedro, February 11, 1949, tide pools
	Gastropoda	*Tegula brunnea* (R. A. Philippi, 1849)	alive	Silén, 1946	Tidepools, Pacific Grove, California, USA
	Gastropoda	*Tegula ligulata* (Menke, 1850)	alive and dead	Soule, 1950	California, Whites Point, San Pedro, January 27, 1949, tide pools. Portuguese Bend, March 11, 1949; tide pools
	Gastropoda	*Haliotis cracherodii* (Leach,1814)	dead	Soule, 1950	Whites Point, San Pedro, January 27, 1949; tide pools
	Gastropoda	*Littorina keenae* (Rosewater, 1978)	dead	Soule, 1950	Whites Point, San Pedro, January 27, 1949; tide pools
	Gastropoda	*Littorina scutulata* (Gould,1849)	dead	Soule, 1950	Whites Point, San Pedro, January 27, 1949; tide pools
cheongpodensis		Mollusc shells; oysters and clams	dead	Seo et al., 2018	Low intertidal zone. Cheongpodae, Taean Coast National Park (36.6334° N, 126.2997° E). 16 June 2017.
orbignyana	*Gastropoda*	*Conus ventricosus* (Gmelin, 1791)	-	Fischer, 1866	Arcachon basin (Gironde), France
	Gastropoda	*Charonia lampas* (Linnaeus, 1758)	-	Fischer, 1866	Arcachon basin (Gironde), France
	Bivalvia	*Ostrea edulis* (Linnaeus, 1758)	-	Fischer, 1866	Arcachon basin (Gironde), France
	Bivalvia	*Unidentified bivalve*	dried	Pohowsky, 1978	Arcachon basin (Gironde), France; Fischer material
I. patagoniana	*Gastropoda*	*Buccinanops cochlidium* (Dillwyn, 1817)	dried	Pohowsky, 1978	Patagonia, Argentina
	Gastropoda	*Argeneuthria cerealis* (Rochebrune & Mabille, 1885)	dead	López-Gappa & Zelaya (2021), Johnson et al.	Burdwood Bank<br>-30 Mar 2016, 54°25.144' S, 59°12.892' W depth 120<br>-8 May 2017 54°00.240' S,61°04.762' W, depth 139<br>-10 Apr 2016, 54°30.390' S, 59°48.654' W, depth 105<br>-31 Mar 2016, 54°31.679' S, 61°27.979' W, depth 137
	Gastropoda	*Cerithiopsis caelata* (Gould, 1849)	dead	López-Gappa & Zelaya (2021)	
	Gastropoda	*Fuegotrophon pallidus* (Broderip, 1833)	dead	López-Gappa & Zelaya, 2021	
	Gastropoda	*Pareuthria atrata* (E. A. Smith, 1881)	dead	López-Gappa & Zelaya (2021), Johnson et al.	
	Gastropoda	*Pareuthria fuscata* (Bruguière, 1789)	alive, dead	López Gappa, 1981, López-Gappa & Zelaya (2021), Johnson et al., this study	
	Gastropoda	*Savatiera chordata* (Castellanos, Rolán & Bartolotta, 1987)	dead	Johnson et al.	
	Gastropoda	*Trophon ohlini* (Strebel, 1904)	dead	Lopez-Gappa & Zelaya (2021), Johnson et al.	

	Gastropoda	*Typhlodaphne* sp. (Powell, 1951)	dead	Johnson et al.	
I. stephanieae	*Gastropoda*	*Littorina littorea* (Linnaeus, 1758)	alive	Johnson et al.	Intertidal zone around the Roscoff Marine Station 48°43.698'N, 3°59.721'W and Santec 48°42.787' N, 4°1.315' W<br>-intertidal<br>Îll Callot, intertidal
	Gastropoda	*Nucella lapillus* (Linnaeus, 1758)	alive	Johnson et al.	Intertidal zone around the Roscoff Marine Station
	Polyplacophora	*Acanthochitona* sp. (J. E. Gray, 1821)	alive	This study	Intertidal zone around the Roscoff Marine Station
I. suecica	*Bivalvia*	*Pseudamussium peslutrae* (Linnaeus, 1771)	alive, dead	Silén (1947)	Gullmar Fjord, West Coast of Sweden, north of Flatholmen, depth 45 m
I. cf. *suecica* France	*Bivalvia*	*Aequipecten opercularis* (Linnaeus, 1758)	dead	This study	Stolvezen, Roscoff 48°42.847' N, 3°53.500' W;<br>48°42.846' N, 3°53.5' W; 48°40.000' N, 3°52.999' W, depth15 – 25<br><br>Térenéz, Roscoff 48°41.532' N, 03°52.075' W, depth ≈10<br><br>Chateaux du Taureau, Roscoff 48°40.2' N, 3°53.12' W;<br>48°40.200' N, 3°52.999' W, depth 10 – 15 m<br><br>Primel, Roscoff 48°43.467'N, 03°50.55'W<br><br>Pleine du Vezael, Roscoff 48°42.48'N, 003°55.33'W<br><br>Near L'lle Louët, and the Roscoff Harbour
	Bivalvia	*Acanthocardia echinata* (Linnaeus, 1758)	dead	This study	
	Bivalvia	*Anomia ephippium* (Linneaus, 1758)	dead	This study	
	Gastropoda	*Buccinum undatum* (Linnaeus, 1758)	dead	This study	
	Gastropoda	*Crepidula fornicata* (Linnaeus, 1758)	dead	This study	
	Bivalvia	*Ensis* sp. (Schumacher, 1817)	dead	Johnson et al., This study	
	Bivalvia	*Glycymeris* (Linnaeus, 1758)	dead	Johnson et al., This study	
	Bivalvia	*Lutraria lutraria* (Linnaeus, 1758)	dead	Johnson et al., This study	
	Bivalvia	*Mimachlamys varia* (Linnaeus, 1758)	dead	This study	
	Bivalvia	*Pecten maximus* (Linnaeus, 1758)	dead	Johnson et al., This study	
	Bivalvia	*Polititapes aureus* (Gmelin, 1791)	dead	This study	
	Gastropoda	*Tritia reticulata* (Linnaeus, 1758)	dead	This study	
I. cf. *suecica* Norway	*Gastropoda*	*Buccinum undatum* (Linnaeus, 1758)	dead, hermited	Johnson et al., This study	
I. zelandica	*Gastropoda*	*Buccinulum littorinoides* (Reeve, 1846)	alive	Silén (1946)	Slipper Island, New Zealand, intertidal
I. cf. *zelandica*	*Gastropoda*	Buccinoidea Rafinesque, 1815, Molluscan shells,	dead	Johnson et al.	Otago inner shelf, New Zealand (45° 45.87' S, 170° 49.50' E). Collected 5 November 2021, depth 40 m
I. zelandica var *minuta*	*Gastropoda*	*Conus striatus* (Linnaeus, 1758)	-	Soule & Soule (1969)	Hawaiian Island, Haena Bay, Kauai; collected 1 July 1967, depth 20 feet (6.1 m),<br>water temperature 80°F
	Gastropoda	*Cypraea* sp.? (Linnaeus, 1758)	-	Soule & Soule (1969)	
	Gastropoda	*Stomatella planulata* (Lamarck, 1816)	alive	Soule 1950	Zamboanga, Philippine Islands, tide pools
Immergentia spp. Guam	*Gastropoda*	*Conus* sp. (Linnaeus, 1758)	dead, hermited	This study	Pago Bay (13°25'36.9"N, 144°47'41.8"E), area behind University of Guam Marine Lab, intertidal, 13 & 15 November 2022
	Gastropoda	*Morula uva* (Röding, 1798)	alive	This study	

	Gastropoda	*Fusinus colus* (Linnaeus, 1758)	dead	This study	Family Beach (13°27'41.7"N, 144°38'51.1"E), Guam, depth 7 m
Immergentia spp. Japan	*Gastropoda*	Shell fragment	dead	This study	Sagami Bay west,<br>Manazuru, Japan (35°8'29.03"N, 139°9'39.74"E), collected 19 August 2023, intertidal
					Ise Bay area, Japan<br>Dredge: Enshu Sea (34°17'60.00"N, 137°5'45.00"E-34°18'4.20"N, 137°6'24.60"E), depth 153-154 m<br><br>Beam Trawl: Enshu Sea (34°17'51.60"N, 137°7'16.80"E-34°17'45.00"N, 137°8'7.20"E), depth 165-180 m<br>Both collected 23 August 2023
					Sagami Bay, east<br>Tenjin-jima, Japan (35°13'16.82"N, 139°36'10.56"E), collected 29 August 2023, intertidal
Immergentia spp.	*Gastropoda*	*Buccinulum corneum* (Linneaus, 1758)	dried	This study*	Syracuse, Sicily, Italy<br>Depth not reported (sample no. G860.40.10.1)
	Gastropoda	*Bursa thomae* (d'Orbigny, 1842)	dried	This study*	Tenerife, Canary Islands (South of Teno Point)<br>20 - 25m depth (sample G790.10.40.1; G790.10.40.2)
	Bivalvia	*Chlamys islandica* (Müller, 1776)	dried	This study*	Ramfjorden, Tromsø, Norway (N69.308-E10.567)<br>119m depth
	Bivalvia	*Chlamys islandica* (Müller, 1776)	dried	This study*	Bay of Biscay-Penmarc'h, France (N46.9043-W5.4432)<br>610m depth (sample G790.10.40.1; B190.130.40.9, SaM77947)
	Gastropoda	*Ocenebra brevirobusta* (Houart, 2000)	dried	This study*	Moroccan Coast 15km North of Agadir. Intertidal (sample G860.90.5.1)
	Gastropoda	*Tritia reticulata* (Linnaeus, 1758)	dead	This study	Roscoff, intertidal zone
	Bivalvia	*Polititapes aureus* (Gmelin, 1791)	dead	This study	See locations for *I.* cf. *suecica* France
	Bivalvia	*Pseudamussium clavatum* (Poli, 1795)	dried	This study*	Bay of Biscay-Penmarc'h, France (N46.9043-W5.4432)<br>610m depth (sample G790.10.40.1; B190.80.10.5, SaM70699)
	Gastropoda	Unidentified gastropod	dead	This study*	Guadeloupe; between the south of Basse-Terre and Marie-Galante, Caribbean Sea (15°58'00.0"N, 61°25'50.0"W), 55m depth, Poseidon cruise 316, SAM ID-15820

Note: Substrate species names according to WoRMS classification

(*) = Senckenberg am Meer collection

Table S2. Characteristics of immergentiids from new locations examined in this study.

**Location *Immergentia* Species**	**Mean aperture width (µm)**	**Borehole aperture shape**	**Colony + zooidal characteristics**
Guam	51.07 ± SD 12.96<br><br>Min - max: 26.37 – 76.95	Oval to spindle-shaped	Only one shell left<br><br>Densely packed difficult to distinguish colony pattern.
	n = 30		
Helgoland	47.176 ± SD 9.61<br><br>Min - max: 31.98 – 57.66	Circular to oval-shaped	Densely packed difficult to distinguish colony pattern.
	n = 15		
Japan	51.34 ± SD 7.45<br><br>Min - max: 41.95 – 70.56<br><br>Ancestrula width 40.49	Circular to oval-shaped	Distinct feather-like pattern of colony.<br><br>Lateral cystid appendages always extend opposite each other if two are present.
	n = 14		
Guadeloupe	39.67 ± SD 5.8<br><br>Min - max: 30,39 – 48.5	Circular	Colony form not determined (cystid appendages not visible on shell)<br><br>Zooids typical vase shape with rounded or pointed tip. Sometimes tip tilted in direction of primary cystid appendage.
	n = 11		Zooid length<br>296.56 ± SD 30<br>Min - max: 257.92 – 362.96<br><br>Zooid width<br>64.04 ± SD 9.55<br><br>Min - max: 47.06 – 81.50<br><br>n = 10

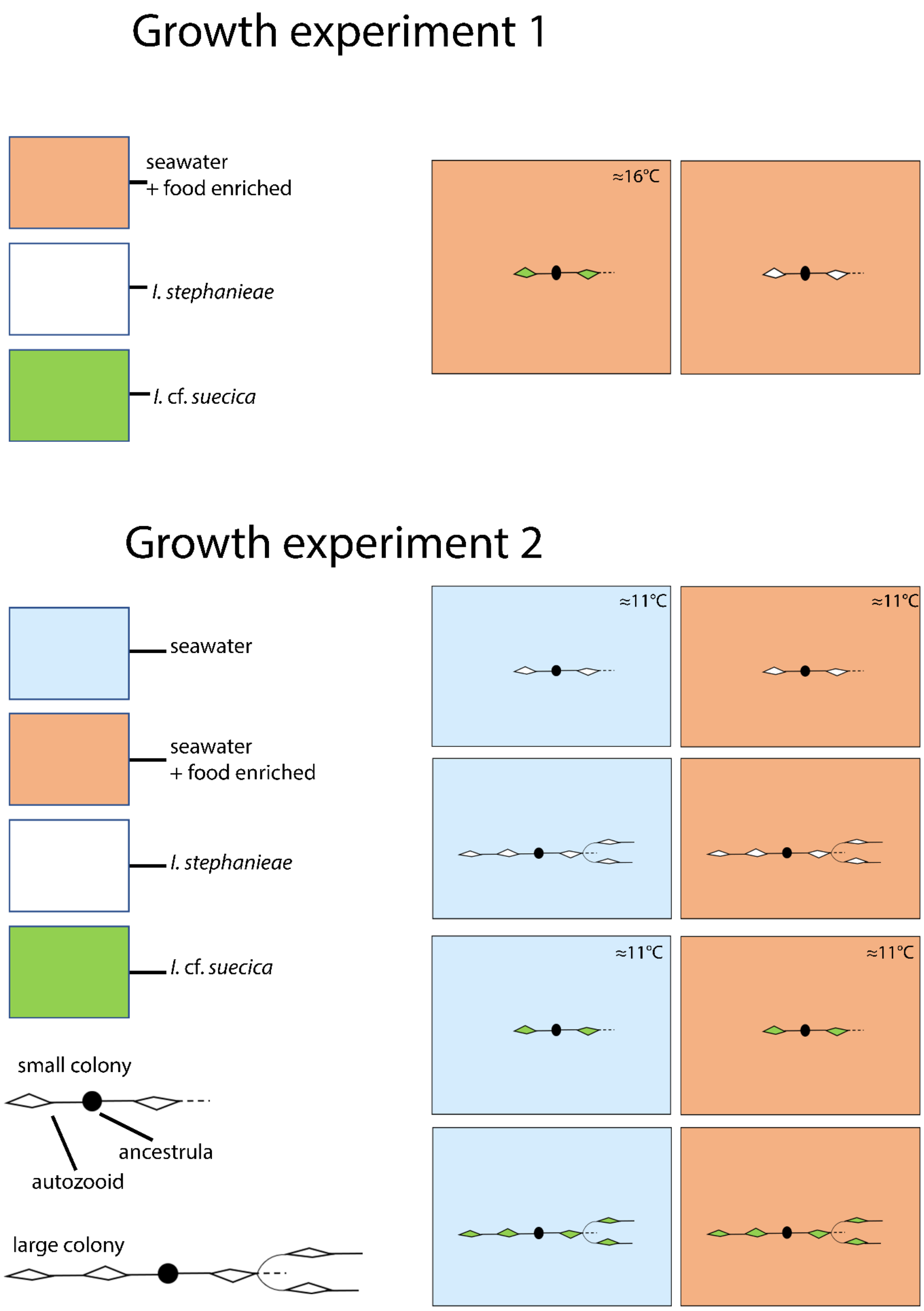

Figure S1. Experimental design of growth experiments 1 occurred in the period between 16 August to 16 October 2021 and growth experiment 2 between 27 February to 22 March 2023.

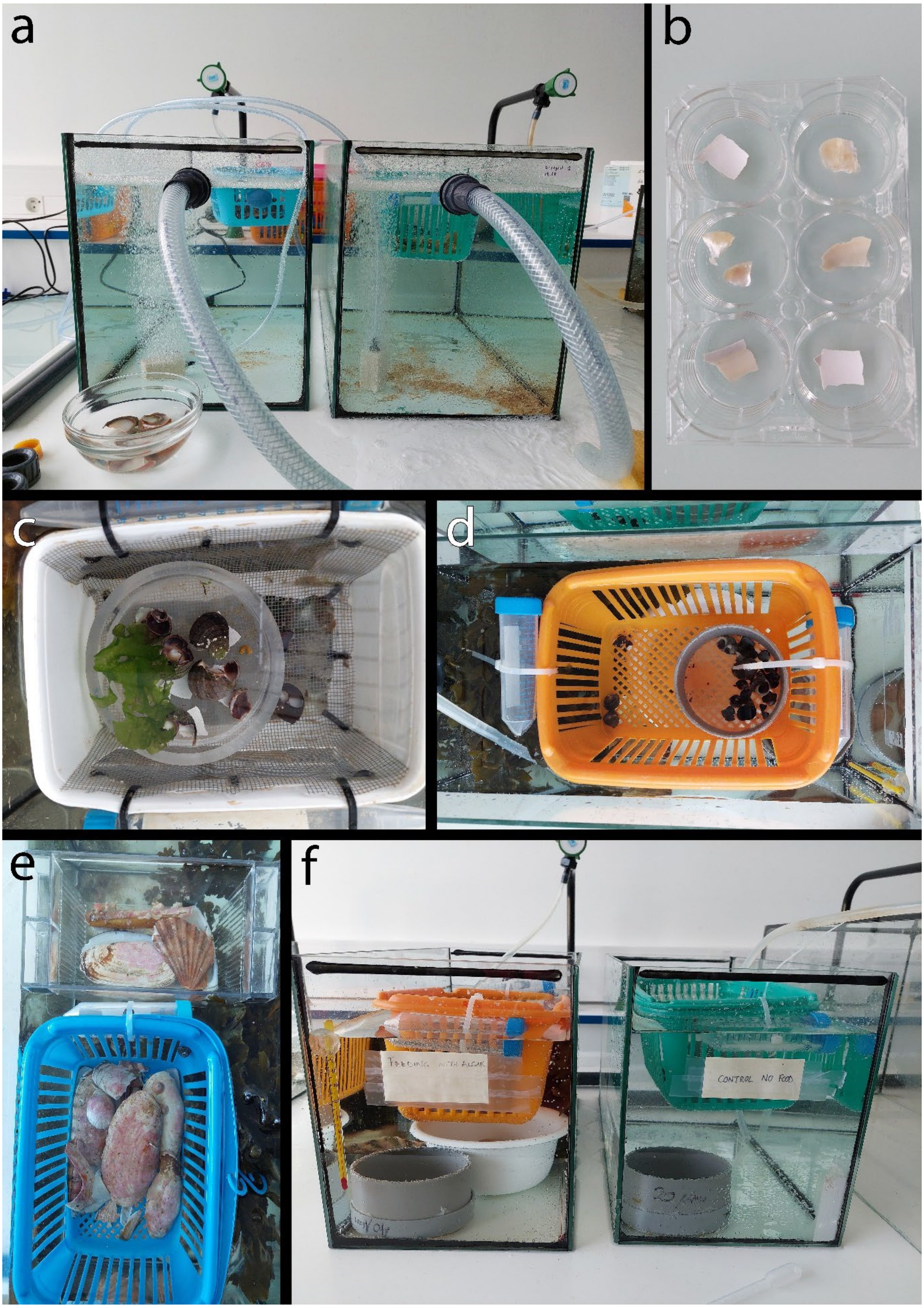
a
b
c
d
e
f
FEEDING WITH ALGAE
CONTROL NO FOOD

Figure S2. **a** Experimental set up for growth experiment 1 from 16 August to 16 October 2021. **b** Nunc 6-well multidishes for settlement experiment 1A with shells uncolonized molluscan shells. **c** Gastropod shells with colonies placed on uncolonized molluscan shells in a disk with fine mesh for settlement experiment 1B. **d – f** Settlement experiment 2 from 27 February to 22 March 2023. **d** *Littorina littorina* bearing *I. stephanieae* colonies placed with uncolonized shells for settlement experiment 2B. **e** bivalve shells with *I.* cf. *suecica* shells placed on uncolonized bivalve shells for settlement experiment B. **f** Growth and settlement experiment 2A set up with food enriched and not enriched (control) tanks.

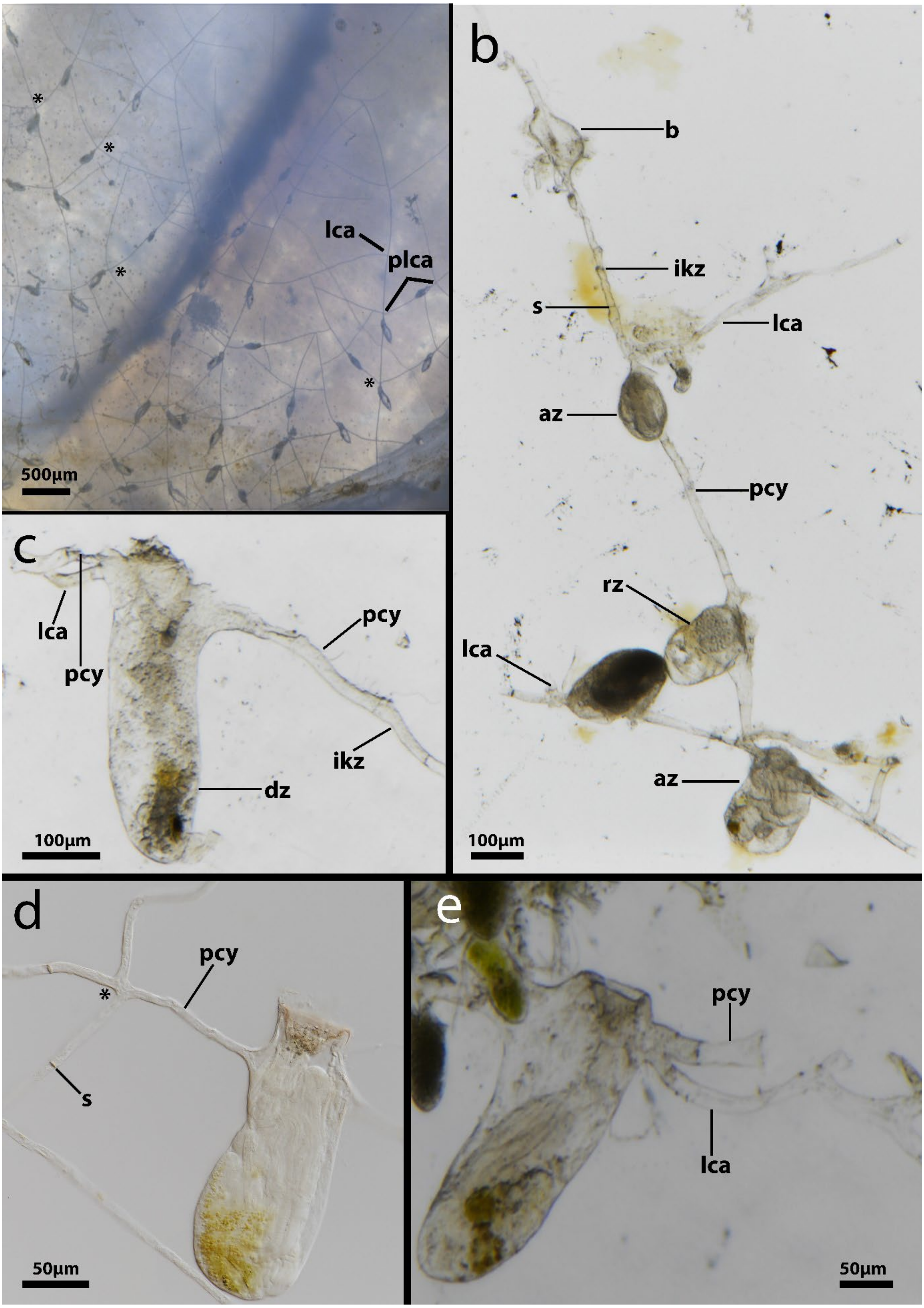

b
c
d
e
lca
plca
500µm
b
ikz
s
lca
az
pcy
rz
lca
az
100µm
lca
pcy
pcy
ikz
dz
100µm
pcy
s
50µm
pcy
lca
50µm

Figure S3. **a** *Immergentia* cf. *suecica* colony with paired lateral cystid appendages (asterisks) creating a feather-like pattern and lateral cystid appendages. **b** Part of *Immergentia stephanieae* colony with zooids in different developmental stages. Lateral cystid appendages extend from the primary cystid appendage. Intercalary kenozooids with septum separate cystid appendages of neighbouring zooids. **c** Lateral cystid appendage developed near the primary cystid appendage in *Immergentia stephanieae* from Roscoff, France. **d** Primary cystid appendage spit, septa develop after and paired lateral cystid appendages developed (asterisk) in *Immergentia* cf. *zelandica* from Otago Inner shelf, New Zealand **e** Lateral cystid appendage derived from cystid wall developed near the primary cystid appendage *Immergentia stephanieae*.

Abbreviations: anc – ancestrula, az – autozooid, b – bud, d – degenerated zooid, ikz – intercalary zooid, lca – lateral cystid appendage, pcy – primary cystid appendage, plca – paired lateral cystid appendage, rz – reproductive zooid, s – septum

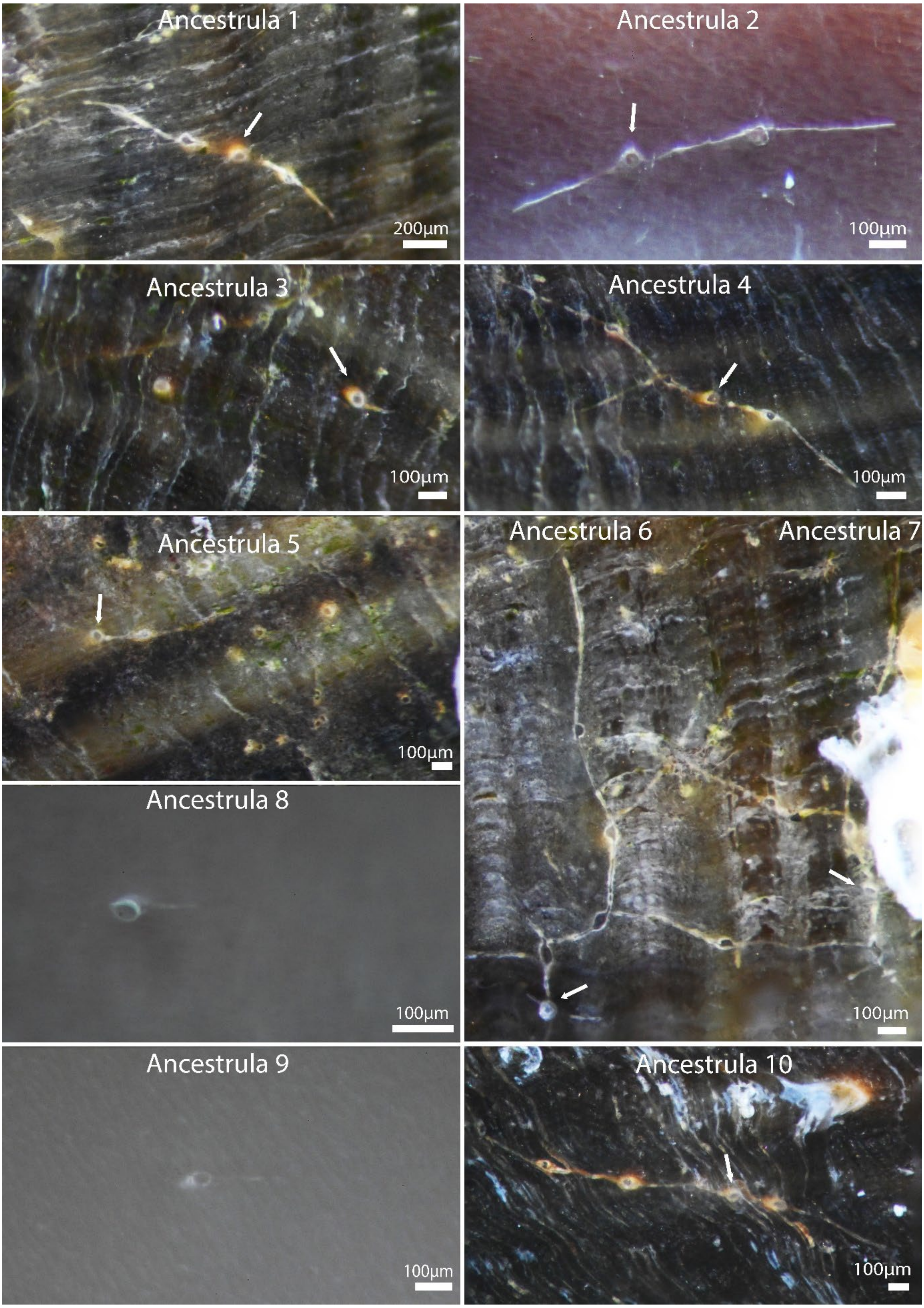

Ancestrula 1
200µm
Ancestrula 2
100µm
Ancestrula 3
100µm
Ancestrula 4
100µm
Ancestrula 5
100µm
Ancestrula 6
Ancestrula 7
100µm
Ancestrula 8
100µm
Ancestrula 9
100µm
Ancestrula 10
100µm

Figure S4. Ancestrulae and small colonies of *I. stephanieae* from Growth experiment 1 corresponding to Table 1 in the main text. Images on final day of observation.

Video S1. Flickering and caging of tentacles by *Immergentia stephanieae*

figshare private link: https://figshare.com/s/3b410cfc1ffe4a7b152c

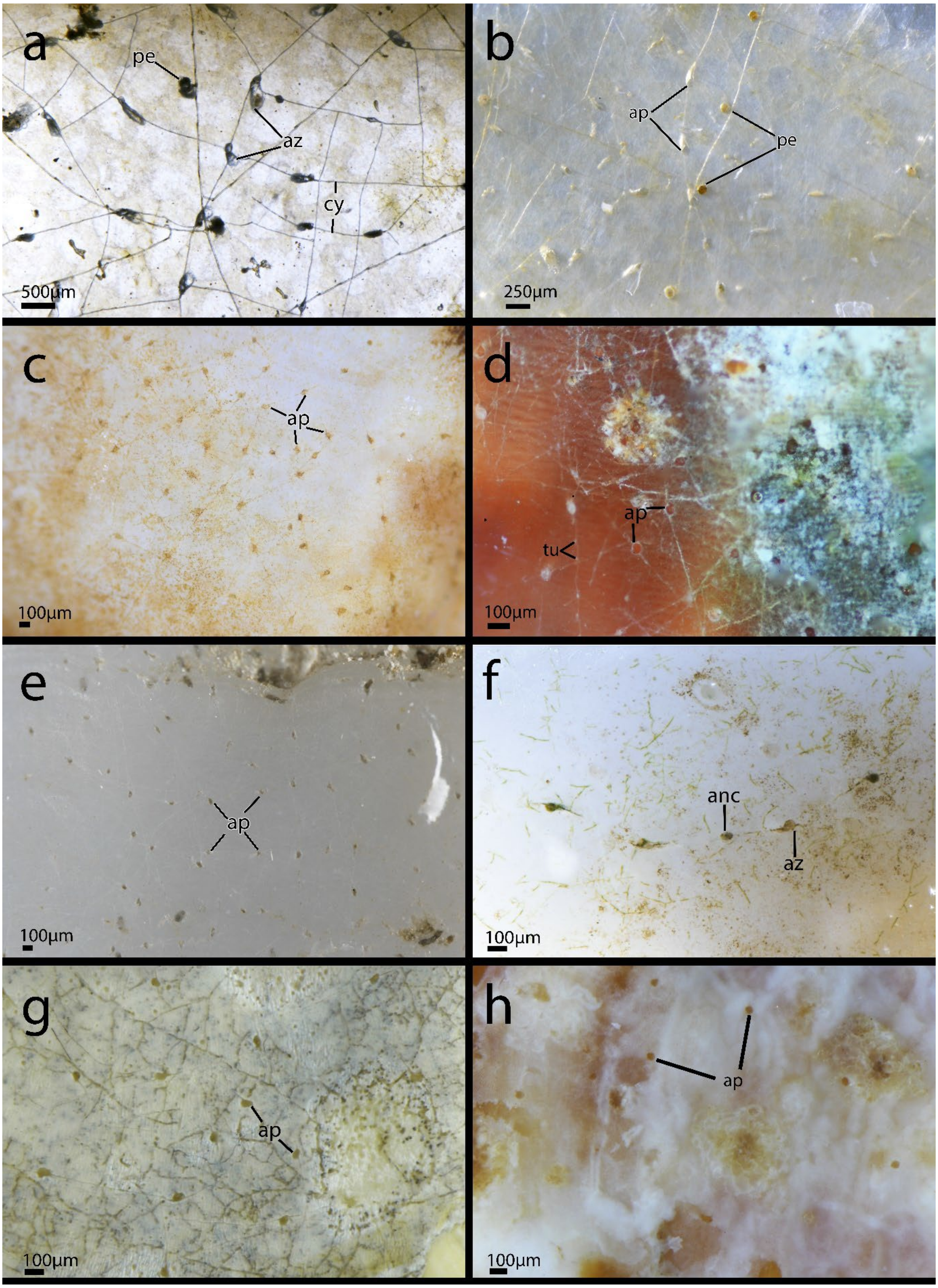

a
pe
az
cy
500µm
b
ap
pe
250µm
c
ap
100µm
d
ap
tu
100µm
e
ap
100µm
f
anc
az
100µm
g
ap
100µm
h
ap
100µm

Figure S5. Colonies of immergentiids reported from new locations. **a** Colony of unidentified *Immergentia* sp. from Helgoland in an *Anomia* sp. shell. Autozooids and cystid appendages visible. **b** Same colony. Circular-shaped borehole apertures of immergentiid. Pedunculate *Penetrantia* sp. also visible. **c – e** *Immergentia* sp. from Guam with spindle-shaped borehole apertures. **c** Immergentiid in hermited *Conus* sp. shell collected from Pago Bay. **d** Immergentiid in gastropod *Morula uva* shell collected from Pago Bay with circular boreholes. **e** Immergentiid in shell of *Fusinus colus* collected from Family Beach. **f** Small immergentiid colony from Sagami area (Japan) with boreholes of ancestrula and two autozooids on either side in gastropod shell. Autozooids directly next to ancestrula extending at 180° angle from ancestrula. **g** Overview of immergentiid colony from Japan. **h** Colony from the Caribbean Sea (Guadeloupe) with circular borehole apertures.

Abbreviations: anc – ancestrula, ap – aperture, az – autozooid, cy – cystid appendage, pe – *Penetrantia*, tu – tubulets
